# The Effects of a Lifestyle Intervention Supported by the InterWalk Smartphone App on Increasing Physical Activity Among Persons With Type 2 Diabetes: Parallel-Group, Randomized Trial

**DOI:** 10.2196/30602

**Published:** 2022-09-28

**Authors:** Ida Kær Thorsen, Yanxiang Yang, Laura Staun Valentiner, Charlotte Glümer, Kristian Karstoft, Jan Christian Brønd, Rasmus Oestergaard Nielsen, Charlotte Brøns, Robin Christensen, Jens Steen Nielsen, Allan Arthur Vaag, Bente Klarlund Pedersen, Henning Langberg, Mathias Ried-Larsen

**Affiliations:** 1 Center of Inflammation and Metabolism and Centre for Physical Activity Research Copenhagen University Hospital - Rigshospitalet Copenhagen Denmark; 2 Chair of Sport and Health Management Technical University of Munich Munich Germany; 3 CopenRehab Department of Public Health University of Copenhagen Copenhagen Denmark; 4 Centre for Diabetes Municipality of Copenhagen Copenhagen Denmark; 5 Department of Clinical Pharmacology Bispebjerg Hospital Copenhagen Denmark; 6 Research Unit for Exercise Epidemiology Centre of Research in Childhood Health Department of Sports Science and Clinical Biomechanics, University of Southern Denmark Odense Denmark; 7 Department of Public Health Aarhus University Aarhus Denmark; 8 Research Unit for General Practice Aarhus University Aarhus Denmark; 9 Department of Endocrinology Diabetes and Bone-metabolic Research Unit Rigshospitalet Copenhagen Denmark; 10 Steno Diabetes Center Copenhagen Gentofte Denmark; 11 Section for Biostatistics and Evidence-Based Research The Parker Institute Bispebjerg and Frederiksberg Hospital Copenhagen Denmark; 12 Research Unit of Rheumatology Department of Clinical Research University of Southern Denmark, Odense University Hospital Odense Denmark; 13 Danish Centre for Strategic Research in Type 2 Diabetes Steno Diabetes Center Odense Odense University Hospital Odense Denmark

**Keywords:** type 2 diabetes mellitus, exercise, telemedicine, primary health care, accelerometry, quality of life, waist circumference, mHealth, mobile app

## Abstract

**Background:**

Effective and sustainable implementation of physical activity (PA) in type 2 diabetes (T2D) health care has in general not been successful. Efficacious and contemporary approaches to support PA adherence and adoption are required.

**Objective:**

The primary objective of this study was to investigate the effectiveness of including an app-based (InterWalk) approach in municipality-based rehabilitation to increase moderate-and-vigorous PA (MVPA) across 52 weeks compared with standard care among individuals with T2D.

**Methods:**

The study was designed as a parallel-group, randomized trial with 52 weeks’ intervention and subsequent follow-up for effectiveness (52 weeks from baseline). Participants were recruited between January 2015 and December 2016 and randomly allocated (2:1) into 12 weeks of (1) standard care + InterWalk app–based interval walking training (IWT; IWT group; n=140), or (2) standard care + the standard exercise program (StC group; n=74). Following 12 weeks, the IWT group was encouraged to maintain InterWalk app–based IWT (3 times per week for 30-60 minutes) and the StC group was encouraged to maintain exercise without structured support. Moreover, half of the IWT group (IWTsupport group, n=54) received additional motivational support following the 12-week program until 52-week follow-up. The primary outcome was change in objectively measured MVPA time (minutes/day) from baseline to 52-week follow-up. Key secondary outcomes included changes in self-rated physical and mental health–related quality of life (HRQoL), physical fitness, weight, and waist circumference.

**Results:**

Participants had a mean age of 59.6 (SD 10.6) years and 128/214 (59.8%) were men. No changes in MVPA time were observed from baseline to 52-week follow-up in the StC and IWT groups (least squares means [95% CI] 0.6 [–4.6 to 5.8] and –0.2 [–3.8 to 3.3], respectively) and no differences were observed between the groups (mean difference [95% CI] –0.8 [–8.1 to 6.4] minutes/day; *P*=.82). Physical HRQoL increased by a mean of 4.3 (95% CI 1.8 to 6.9) 12-item Short-Form Health Survey (SF-12) points more in the IWT group compared with the StC group (Benjamini-Hochberg adjusted *P*=.007) and waist circumference apparently decreased a mean of –2.3 (95% CI –4.1 to –0.4) cm more in the IWT group compared with the StC group but with a Benjamini-Hochberg adjusted *P*=.06. No between-group differences were observed among the remaining key secondary outcomes.

**Conclusions:**

Among individuals with T2D referred to municipality-based lifestyle programs, randomization to InterWalk app–based IWT did not increase objectively measured MVPA time over 52 weeks compared with standard health care, although apparent benefits were observed for physical HRQoL.

**Trial Registration:**

ClinicalTrials.gov NCT02341690; https://clinicaltrials.gov/ct2/show/NCT02341690

## Introduction

Physical activity (PA) is a cornerstone in the prevention and management of type 2 diabetes (T2D) [1] and adults with T2D are recommended to perform a minimum of 150 minutes of moderate-and-vigorous PA (MVPA) per week [2]. However, effective and sustainable implementation of PA programs in health care has in general not been successful [3-7]. These barriers may include low self-efficacy, inappropriate goal-setting, lack of access to facilities, and lack of supervision and social support [2]. Previous efforts to support increased PA levels among individuals with T2D have produced promising results in trial contexts, but the extensive support applied may involve limited longer-term effectiveness of these efforts [8-12]. Accordingly, sustained increases in PA levels are rarely reported [10-15]. Exercise supervision may be required to improve glycemic control among individuals with T2D, whereas advice alone is insufficient [16]. Altogether, continued support to sustain PA levels is pivotal. Given the growing global prevalence and incidence of T2D [17], providing fully supervised exercise on a life-long basis is unfeasible. Thus, efficacious and contemporary approaches to support PA adherence are required.

While MVPA is recommended for individuals with T2D [1,2], brief high-intensity exercise bouts may also be effective in increasing physical fitness and improving glycemic control [18]. Accordingly, we have previously shown that 4 months of technology-supported interval walking training (IWT; 5 sessions of 60 minutes/week) led to increased physical fitness, decreased body mass and adiposity, and improved glycemic control among individuals with T2D, whereas energy expenditure–matched continuous walking did not [19]. In addition to promoting increased peak intensities of PA, IWT is a safe and convenient exercise type that has proven effective in maintaining adherence and motivation to continue IWT after a trial, especially when receiving feedback from a training device [20].

The increasing implementation of digital solutions in health care [1,21], along with the growing smartphone ownership among the older populations (≥50 years) [22], suggests the use of smartphones as an easy, accessible opportunity for remote and flexible PA support. Accordingly, the InterWalk app for smartphones was developed in Denmark to deliver individually tailored IWT as a feasible intervention to promote PA among individuals with T2D [20,23]. Further, we have previously observed that motivational support consisting of goal setting, SMS text message support, and phone calls increased adherence to InterWalk app–based IWT [24].

The primary aim of this study was to test the hypothesis that InterWalk app–based IWT implemented in a municipality-based health care setting is superior in increasing MVPA across 52 weeks compared with standard care among individuals with T2D. Secondarily, we investigated the effects of the intervention on self-rated physical and mental health–related quality of life (HRQoL), physical fitness, weight, and waist circumference. Moreover, we wanted to explore the effects of additional motivational support for InterWalk app–based IWT on these outcomes.

## Methods

### Study Design

The study was a parallel-group, randomized trial with 52 weeks of intervention and subsequent effectiveness follow-up a year after baseline. All participants provided oral and written informed consent prior to commencing any study procedures. The original protocol has been published [23], while the prespecified statistical analysis plan is available as Multimedia Appendix 1. Reporting is in accordance with the CONSORT statement.

### Ethics Approval

The study was approved by the scientific ethical committee of the Capital Region of Denmark (H-1-2014-074) and registered at clinicaltrials.gov (NCT02341690).

### Participants and Eligibility

Inclusion criteria were T2D diagnosis, ≥18 years of age, and referral to a municipality health promotion center or hospital in the participating municipality by the individual’s general practitioner. Exclusion criteria were medical contraindications to exercise, for example, chronic complications in the musculoskeletal system, painful osteoarthritis, or heart conditions [25]; declining to participate in an exercise program at the health promotion center or hospital; current participation in other intervention studies; or insufficient Danish language skills. All individuals who met for an appointment at the participating health promotion centers or hospital were screened through medical records and at a screening interview with a health professional at the center or hospital.

The data were collected at the participating health promotion centers (Amager, Vanløse, Østerbro, Vesterbro in the Municipality of Copenhagen, Denmark; and Municipality of Guldborgsund, Denmark) and a hospital (Bornholm, Denmark).

### Randomization and Blinding

Participants were randomly allocated (2:1) into 1 of 2 arms: (1) standard care + InterWalk app–based IWT (IWT group) or (2) standard care + the standard exercise program (StC group). Following the initial 12-week supervised exercise program, the participants in the IWT group were further randomly allocated (1:1) into (1) IWT, no additional support (IWT_only_ group) or (2) IWT, with additional support (IWT_support_ group), that is, participants in the IWT_only_ group and the IWT_support_ group underwent similar interventions during the 12-week exercise program, and allocation to either of these was concealed until after the 12-week intermediate assessment.

Participants were randomized using random permuted blocks stratified by sex (2 levels) and center (6 levels). The allocation sequence was generated through a standardized computer program by an independent statistician (RC) and stored on a password-protected computer by an independent researcher (RN) who was not involved in any study procedures. Following the completion of all baseline measurements, the independent researcher was contacted and performed allocation (to StC or IWT_only_ or IWT_support_). The respective group allocation for the initial 12 weeks (StC or IWT) was subsequently returned by email to the health professional who informed the participant about the allocation by telephone call. Information about IWT_only_ or IWT_support_ was not disclosed by the independent researcher to the health professionals until week 12. Following the 12-week intermediate assessment, the independent researcher was contacted and the allocation into IWT_only_ or IWT_support_ was returned by email to the health professional who informed the participant about the allocation by telephone call. The health professionals carried through the data collection and intervention, and thus, were only blinded to the primary outcome.

### Interventions

#### Development and Implementation

The interventions have been described in detail elsewhere [23]. Briefly, the interventions were designed to comply with the standard health care in Denmark [26]. Study investigators (LV, CB, and HL) prepared and led several workshops totally 16 hours), where the health professionals and the investigators discussed the normal work routines in detail and discussed suggestions on how to implement the InterWalk app and co-interventions into the daily routine. Based on these discussions, the intervention protocol was developed. Following the finalization of the study protocol, the health professionals completed an educational program (15 hours in total), where they were trained in implementation of the study procedures and manuals. In addition, the health care professionals attended workshops every second month throughout the trial period to ensure the procedures and manuals were consistently implemented as described in the protocol.

#### Baseline to 12-Week Follow-up

As part of normal practice, lifestyle interventions may be prescribed to individuals with T2D by their general practitioner by referral to municipality-based lifestyle programs [26]. This program entails a combination of exercise, diabetes education on self-management, smoking cessation courses, and diet counseling. A patient might receive all or any combination of these components, depending on the specific need of the patient. The decision is based on a dialog between the patient and the health care provider upon initiation of the program.

Both the IWT and StC groups underwent 12 weeks of standard municipality-based health care [23,26]. They both received an exercise program during the initial 12 weeks. The StC group was prescribed a standard municipality-based exercise program (2 sessions per week for 12 weeks; combined aerobic and resistance training delivered by trained health professionals). The IWT group was prescribed InterWalk app–based IWT (30-60 minutes per session, 3 sessions per week for 12 weeks; for an extended description, see below) instead of the standard exercise program. During the 12-week exercise program, IWT was group based and 2 of the 3 sessions were supervised.

#### From 12-Week to 52-Week Follow-up

Following the initial 12 weeks, the StC group was encouraged to maintain exercise without structured support.

The participants allocated to the InterWalk app–based intervention from baseline to 12-week follow-up were either allocated to be encouraged to maintain InterWalk app–based IWT (3 times per week for 30-60 minutes; IWT_only_) until 52-week follow-up or was, for explorative purposes, allocated to additional motivational support following the 12-week program and until the 52-week follow-up (IWT_support_). Feasibility and usability have been described elsewhere [24]. The motivational support included (1) individual motivational interviews with individual goal setting performed by the health care professionals, (2) InterWalk-based IWT with voluntary ambassadors affiliated with the Danish Diabetes Association (a Danish not-for-profit patient organization) once per week, and (3) SMS text message support (once per week).

The motivational interviews were semistructured and performed during weeks 16, 20, 28, and 40 after the baseline assessment. They were designed to structure the communication between the participant and the health professional. The interviews intended to facilitate a partnership formed to reveal and visualize the patients’ motivation and barriers toward the intended behavior change and acknowledgement of patient autonomy [27]. Moreover, the health care professionals were instructed to help the participants perform goals toward a lifestyle change (ie, not only increased PA) using the S.M.A.R.T. principle derived from the Goal Setting Theory [23]. In the S.M.A.R.T. principle S denotes *specific*, M denotes *measurable* (eg, can we track it?), A denotes *achievable* (eg, is it realistic to obtain?), R is for *relevant* (eg, does it make sense for the participant?), and T denotes *timely* (eg, is it obtainable within a prespecified period?).

The ambassador program was a part of a peer-to-peer educational program, where the Danish Diabetes Association offered group walking for all members. The participants were invited to attend these walking groups, where the walking activity was implemented using the InterWalk app.

Finally, facilitation of high adherence to IWT was based on automated feedback to self-reported adherence to IWT using weekly bidirectional SMS text message surveys [24]. An automatic survey was sent during the afternoon on Sundays and inquired about the frequency of IWT during the past week (1=“I have walked more than 3 times/week”, 2=“I have walked 3 times/week”, 3=“I have walked 2 times/week”, 4=“I have walked 1 times/week”, and 5=”I did not walk”). If no answer was received, a reminder was sent within 24 hours. If no answer was received following the reminder or the participant answered, “I did not walk,” the health care professional was instructed to reach the participant by phone using a semistructured approach. The semistructured interview guide consisted of 2 overall questions: (1) Do you experience barriers toward IWT? If yes, which ones? (2) How can I help you to overcome these barriers? If the participant indicated walking 1-2 times/week, an automated SMS text message was sent encouraging the participant to walk more.

#### The InterWalk App

The design and functionality of the InterWalk app was developed in collaboration with persons with T2D using a participatory design. The full details have been described in detail elsewhere [20]. Briefly, following the initial testing of the exercise modality [19], we developed a mock-up of the app and invited persons with T2D to provide their initial feedback. Following the development of the beta-version of the app, 3 iterations of user feedback were incorporated into the design before releasing the version used in this study (versions 8 and 9) [20].

The app was designed to specifically facilitate IWT through continuous individualized audio feedback. The app guides and paces the user through repeated cycles of 3-minute slow walking and 3-minute fast walking [20]. IWT was individualized based on a 7-minute standardized walking test in the InterWalk app [20], which the participants performed at baseline and were asked to repeat every 4 weeks. The intensity was derived from the onboard accelerometer. The individualized cut-offs were determined during the 7-minute walking test and implemented in the IWT sessions. The cut-offs were based on the median intensity between moderate and fast walking pace (the lower limit of intensity during fast IWT walking) and low and moderate walking pace (the upper limit of intensity during slow IWT walking) during the 7-minute walking tests. If participants exceeded these cut-offs during IWT (below the lower limit during fast walking or above the upper limit during slow walking), they received an audio prompt encouraging them to increase or decrease the walking pace. Following each IWT session, the app displayed data from the session performed, such as information about walking distance, steps, duration, and the quality of performance. The latter was determined using the fraction of training time (percentage) which the participant spent within the intensity cut-offs.

Following an IWT session, the objectively measured adherence (intensity, duration, and frequency) to IWT sessions was calculated based on the electronically logged information from the InterWalk app. Data from the InterWalk app were continuously uploaded to a central and secure server throughout the duration of the trial. Self-reported adherence to IWT was calculated based on data from the SMS text message survey, including frequency of weekly use of the InterWalk app and reasons for not using the InterWalk app [24].

### Outcomes

Primary and key secondary outcome measurements are described in further detail in the published protocol [23]. Outcome measurements were conducted by trained health professionals at the respective center or hospital.

The primary outcome measure was change in objectively measured MVPA time (minutes/day) from baseline to 52-week follow-up. PA outcomes were assessed using accelerometers (AX3; Axivity) attached to the participants’ thigh and back using an adhesive tape (Fixomull Stretch; BSN Medical Inc.) and worn for 7-10 consecutive days at baseline, 12 weeks, and 52 weeks. Accelerometer setup and download of raw data (100 Hz) and downsampling to 30 Hz were performed using OmGui (version 1.0.0.28) [28]. The data were then exported to ActiGraph raw binary files (gt3x files) and resampled into counts (agd files) using ActiLife (version 6.11.6). The final data reduction and generation of PA outcome variables were done using a custom-built software developed at the University of Southern Denmark [29]. MVPA time was defined according to the Freedson cut point, ≥1952 counts per minute (CPM) [30], using the vertical axis of the accelerometer placed on the back. Participants were included in the analyses of objectively measured PA if they had ≥3 days of ≥22 hours of measurement (ie, allowing for a 2-hour nonwear time [31]). Accelerometer wear time and nonwear time are presented in Multimedia Appendix 2.

Key secondary outcome measures include changes in physical and mental HRQoL, physical fitness (peak oxygen consumption [VO_2peak_]), self-rated PA energy expenditure (PAEE), exercise motivation, weight, and waist circumference, which were assessed at baseline, 12 weeks, and 52 weeks. Physical and mental HRQoL were assessed using the 12-item Short-Form Health Survey (SF-12), including 8 subscales [32]. The Physical Component Summary (PCS; score 0-100) was calculated based on the 4 subscales: Physical Functioning, Role Physical, Bodily Pain, and General Health; and the Mental Component Summary (MCS; score 0-100) was calculated based on the 4 subscales: Vitality, Social Functioning, Role Emotional, and Mental Health [32]. VO_2peak_ (ml O_2_/minute) was estimated by regression based on the participants’ weight, height, sex, and the acceleration (G) during the last 30 seconds of fast walking in the 7-minute standardized walking test in the InterWalk app [33]. Self-rated PAEE was assessed using the Recent Physical Activity Questionnaire (RPAQ) [34]. Exercise motivation was assessed using the Relative Autonomy Index (RAI) for the Behavioral Regulation in Exercise Questionnaire-2 (BREQ-2) [35]. Weight (kg) was measured using an electronic weight, and waist circumference (cm) was measured midway between the most distal part of the costae and the most proximal part of the iliac crest.

Exploratory secondary outcomes were changes in objectively measured light-intensity PA (100 to <1952 CPM) time (minutes/day), total PA level (CPM), and steps (numbers/day) [30,36] assessed by accelerometers worn on the participants’ back as described above. Sitting time (minutes/day) was derived from the accelerometer placed on the thigh as described elsewhere [31]. BMI (kg/m^2^) was calculated based on weight and height. Any adverse events were reported by the participants to the health professionals at the center or hospital.

### Sample Size

The minimal important difference was considered to be 10 minutes of MVPA per day. Based on existing experimental evidence, we assumed the SD of the change in MVPA time from baseline to 52-week follow-up to be between 1.2 and 2.3 times the difference between groups [37]; accordingly, SD twice the minimal important difference (20 minutes of MVPA per day) was used in the sample size calculations. To maintain a conservative sample size, considering multiple comparisons in a 3-arm trial, Bonferroni adjustment was used in the power calculation. A total of 190 participants were required to achieve a statistical power (1-β) of 80% with an α level of .017 (0.5/3) using an unpaired *t* test (2-sided). Allowing for 30% attrition, 272 participants (91 in the StC group and 181 in the IWT group) should be recruited. The intervention settings enabled recruitment until December 15, 2016, and thus the sample size would include 272 participants or truncated at the number of participants included at the end of the recruitment period—whichever was reached first. On December 15, 2016, 214 participants had been included and they constituted the final analysis population.

### Statistical Methods

A statistical analysis plan was developed and published at the Centre for Physical Activity Research website prior to commencing the statistical analyses [38]; see Multimedia Appendix 1. All continuous outcomes were analyzed using repeated-measures mixed linear models with the baseline value of the relevant variable as a covariate, including fixed effect factors for group 1 (2 levels: IWT/StC), group 2 (3 levels: IWT_only_, IWT_support_, and StC), time (3 levels: baseline, 12-week, 52-week), and group 1 × time interaction; and random effects (patient ID). The analyses were adjusted for the stratifying factors sex (2 levels: male and female) and center (6 levels: Amager, Vanløse, Østerbro, Vesterbro, Guldborgsund, and Bornholm). The following model assumptions were investigated: (1) linearity, (2) normality of residuals, (3) homogeneity of residuals variance, and (4) independence of residual error. Data were analyzed based on the intention-to-treat population (no imputation for missing data) as observed, including all randomized participants with available data at baseline; repeated measures mixed linear models were valid assuming that data were “missing at random.” Results are reported as least squares means (LS means) for each group and the difference between them with 95% CIs. Analyses of categorical outcomes for dichotomous endpoints were performed using the Fisher exact test and reported based on the observed proportions.

To investigate the robustness of the primary analyses, 5 sensitivity analyses were conducted, including 4 analyses of differences in changes in MVPA time from baseline to 52-week follow-up between the IWT group and the StC group: (1) per-protocol analysis, including complete cases of the primary outcome at baseline and 52-week follow-up with ≥70% of the prescribed exercise (3 sessions per week for 52 weeks) completed in the IWT group and no registered IWT sessions in the InterWalk app in the StC group; (2) analysis including all randomized participants with available data at baseline with a conservative single-imputation nonresponder imputation technique (ie, missing data replaced with the baseline observation carried forward); (3) analysis with the subsample of participants not undergoing intervention during a period of InterWalk app malfunctions connected to a major restructuring of the iOS (version 9; available during the period from September 16 to October 21, 2016); and (4) analysis with daytime criteria for inclusion, that is, ≥3 days of ≥14 hours of daytime measurement (6 AM to 22 PM; ie, allowing for 2 hours of nonwear time during this period). In the fifth sensitivity analysis, we used an alternative cut-off at 3000 CPM to investigate changes in forced walking time from baseline to 52-week follow-up between the IWT group and the StC group.

Subgroup analyses were conducted to investigate the effects of IWT versus StC on change in MVPA time (minutes/day) after 52 weeks among subgroups of sex (men/women); T2D duration (≤5 years/>5 years); alcohol consumption (within recommended levels/above recommendations); smoking habits (smoker/nonsmoker); highest level of education (International Standard Classification of Education 2011 [ISCED-2011] levels 0-4/ISCED-2011 levels 5-8); civil status (single, divorced, or widowed/married or cohabiting); baseline SF-12 PCS level (high/low); and baseline SF-12 MCS level (high/low). SF-12 PCS and MCS were divided into high and low using median split. Moreover, differences in median (25th to 75th percentile) duration of IWT (minutes/week) over 52 weeks were investigated among these subgroups.

All reported 95% CIs and *P* values are 2-sided. Because more than 1 comparison was made, key secondary outcome analyses were controlled for the false discovery rate using the Benjamini–Hochberg step-up procedure [39]. All statistical analyses were performed using Stata/IC 13 (StataCorp).

## Results

### Overview

**Figure 1:**
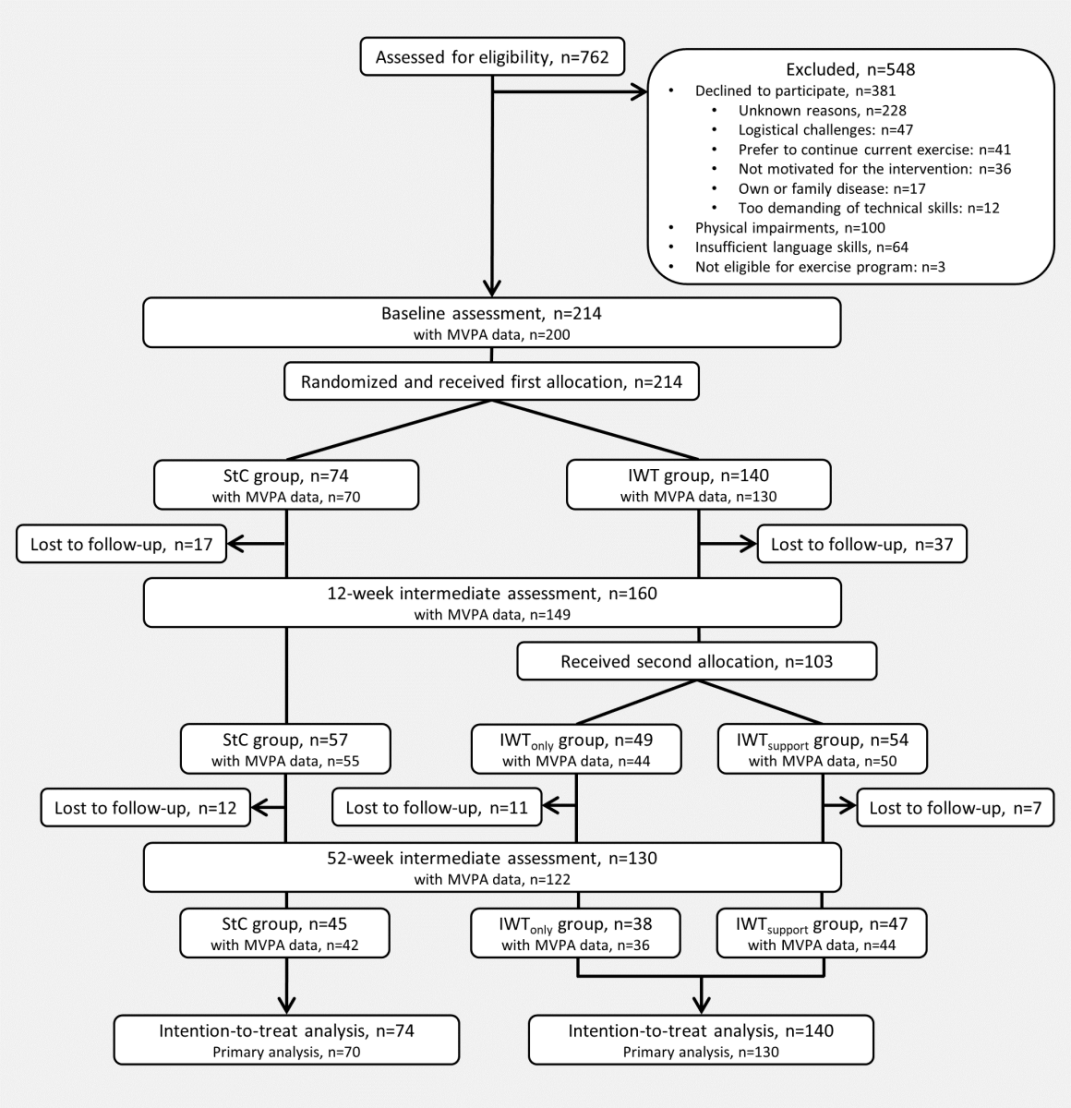
Participant flowchart.

Between January 2015 and December 2016, 762 individuals were screened for inclusion (Figure 1). Of these, 548 were excluded primarily due to declining to participate (n=209) or physical challenges (n=100). Of the 214 participants enrolled in the study, 74 participants were allocated to the StC group and 140 participants to the IWT group. A total of 130 participants completed the 52-week follow-up, which was finalized in December 2017. Participant demographic and clinical characteristics are presented in Table 1. The mean age of participants was 59.6 (SD 10.6) years and 128/214 (59.8%) were men. They had a mean BMI of 34.6 (SD 6.6) kg/m^2^, a median (25th-75th percentile) T2D duration of 2.6 (0.2-8.6) years (Table 1). At baseline, participants spent a median (25th-75th percentile) of 30.2 (16.9-48.1) minutes/week in MVPA (Table 1).

The IWT group engaged in IWT with a median (25th-75th percentile) frequency of 0.3 (0.1-0.7) sessions/week for 11.4 (4.1-22.3) minutes/week at a median intensity of 0.17 (0.11-0.23) G (Multimedia Appendices 3 and 4). For the subgroup that completed the 7-minute standardized walking test in the InterWalk app at baseline, this corresponded to an average intensity across sessions of 65% (SD 49-78) of their maximal intensity at baseline. According to self-reported IWT adherence, participants in the IWT_support_ group used the InterWalk app 1-2 times/week or more in most weeks; the primary reason for not using the InterWalk app was illness (Multimedia Appendix 4). For the IWT_support_ group, 148/188 (78.7%) motivational interviews were completed during follow-up (Multimedia Appendix 5). However, only 27/47 (57%) participants completed all 4 interviews and the adherence to the Danish Diabetes Association walking groups was low as only 5-6/47 (11%-12%) participants participated in this activity. Only a limited number of participants included IWT as a specific part of the goals during follow-up (18/47, 38%).

**Table 1:** Demographic and clinical characteristics of participants at baseline (n=214 patients in the intention-to-treat population).^a^

Demographic and clinical characteristics			StC^b^ group (n=74)		IWT^c^ group (n=140)		Total (n=214)
**Sex**							
	Male	47 (63.5)		81 (57.9)		128 (59.8)	
	Female	27 (36.5)		59 (42.1)		86 (40.2)	
Age (years)			59.8 (10.1)		59.6 (10.8)		59.6 (10.6)
**Type 2 diabetes duration (n=197)**			1.7 (0.2-7.0)		3.0 (0.2-10.0)		2.6 (0.2-8.6)
	≤5 years	45/68 (66.2)		74/129 (57.4)		119 (60.4)	
	>5 years	23/68 (33.8)		55/129 (42.6)		78 (39.6)	
**Alcohol consumption (n=213)**							
	Within the recommended levels	69 (93.2)		129/139 (92.8)		198 (93.0)	
	Above recommendations	5 (6.8)		10/139 (7.2)		15 (7.0)	
**Smoking habits**							
	Smoker	20 (27.0)		29 (20.7)		49 (22.9)	
	Nonsmoker	54 (73.0)		111 (79.3)		165 (77.1)	
**Highest level of education (n=209)**							
	ISCED-2011^d^ levels 0-4	39 (52.7)		75/135 (55.6)		114 (54.5)	
	ISCED-2011 levels 5-8	35 (47.3)		60/135 (44.4)		95 (45.5)	
**Civil status**							
	Single, divorced, or widowed	36 (48.6)		64 (45.7)		100 (46.7)	
	Married or cohabiting	38 (51.4)		76 (54.3)		114 (53.3)	
	Height (cm; n=212)	171.7 (9.4)		172.5 (8.8)		172.2 (9.0)	
**Physical activity and fitness**							
	MVPA^e^ time (minutes/day; n=200)	30.5 (17.3-41.7)		30.2 (16.6-51.8)		30.2 (16.9-48.1)	
	Sitting time (minutes/day; n=195)	556.3 (157.5)		527.3 (162.6)		537.4 (161.0)	
	LPA^f^ time (minutes/day; n=200)	136.3 (52.5)		136.0 (48.7)		136.1 (49.9)	
	TPA^g^ level (CPM^h^; n=200)	212.9 (141.6-291.0)		207.6 (137.8-345.2)		211.5 (140.9-313.8)	
	Steps (n/day; n=200)	4164 (2766-5738)		4078 (2801-6145)		4100 (2794-5986)	
	VO_2peak_^i^ (ml O_2_/minute; n=124)	1859 (511)		1763 (469)		1791 (481)	
**Self-reported measures**							
	SF-12^j^ Physical Component Summary (score 0-100)	40.3 (10.4)		41.5 (10.1)		41.1 (10.2)	
	SF-12 Mental Component Summary (score 0-100)	47.4 (39.6-55.8)		50.9 (41.3-57.1)		50.1 (40.1-56.9)	
	RPAQ^k^ self-rated PAEE^l^ (kJ/kg/day; n=209)	150.1 (92.5-226.7)		136.0 (92.9-225.7)		143.8 (92.9-226.4)	
	BREQ-2^m^ RAI^n^ (score –24 to 20)	7.4 (4.0-10.8)		7.4 (3.5-11.7)		7.4 (3.5-11.3)	
**Body composition**							
	Weight (kg; n=212)	101 (85-119)		98 (87-113)		99 (87-115)	
	Waist circumference (cm; n=211)	117.9 (16.0)		115.3 (12.9)		116.2 (14.1)	
	BMI (kg/m^2^; n=212)	34.6 (6.6)		33.6 (5.3)		34.0 (5.7)	

^a^Data are presented as median (IQR), mean (SD), n (%), or n/N (%).

^b^StC: standard care.

^c^IWT: interval walking training.

^d^ISCED-2011: International Standard Classification of Education 2011.

^e^MVPA: moderate-and-vigorous physical activity.

^f^LPA: light-intensity physical activity.

^g^TPA: total physical activity.

^h^CPM: counts per minute.

^i^VO_2peak_: peak oxygen consumption.

^j^SF-12: 12-item Short-Form Health Survey.

^k^RPAQ: Recent Physical Activity Questionnaire.

^l^PAEE: physical activity energy expenditure.

^m^BREQ-2: Behavioral Regulation in Exercise Questionnaire-2.

^n^RAI: Relative Autonomy Index.

### Change in Moderate-and-Vigorous Physical Activity Time

**Figure 2:**
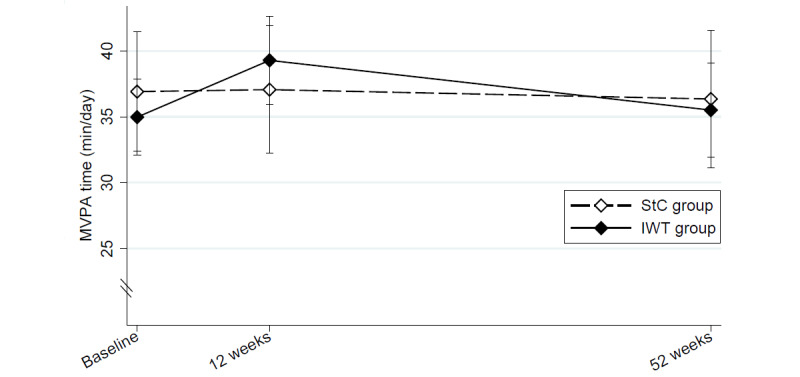
Least squares means (95% CI’s) of MVPA time (min/day) at baseline, 12-week and 52-week follow-up for the IWT and StC group. IWT: interval walking training; MVPA: moderate-and-vigorous physical activity; StC: standard care.

No change in MVPA time was observed from baseline to 52-week follow-up within the StC and IWT groups (*P*=.81 and *P*=.91, respectively; Figure 2) and no between-group difference was observed (–0.8 minutes/day; 95% CI –8.1 to 6.4 minutes/day; *P*=.82; Table 2). From baseline to 12-week follow-up, MVPA increased by a mean of 3.6 (95% CI 0.2 to 6.9) minutes/day in the IWT group and was unchanged in the StC group. However, no difference was observed between the groups (Multimedia Appendix 6).

Only 5 participants in the IWT group fulfilled the per-protocol criteria and the analysis was thus omitted. The remaining sensitivity analyses supported the primary analysis (Multimedia Appendix 7).

**Table 2:** Intention-to-treat analyses of changes from baseline to 52-week follow-up between the IWT group and the StC group^a^.

Outcomes		StC^b^ group (n=74)	IWT^c^ group (n=140)	Between-group difference		
				Difference between means (95% CI)	*P* value	BH^d^ adjusted *P* value
**Primary outcome**						
	MVPA^e^ time (minutes/day; n=200)	0.6 (–4.6 to 5.8)	–0.2 (–3.8 to 3.3)	–0.8 (–8.1 to 6.4)	.82	N/A^f^
	MVPA responders^g^ (n=116)	7/41 (17.1)	17/75 (22.7)	N/A	NA^h^	N/A
**Key secondary outcomes**						
	SF-12^i^ PCS^j^ (score 0-100)	0.0 (–1.9 to 1.8)	4.3 (3.1 to 5.6)	4.3 (1.8 to 6.9)	.001	.007
	SF-12 PCS responders^g^ (n=128)	15/45 (33.3)	45/83 (54.2)	N/A	NA	N/A
	SF-12 MCS^k^ (score 0-100)	2.6 (0.2 to 5.0)	1.3 (–0.4 to 2.9)	–1.3 (–4.7 to 2.0)	.43	.43
	SF-12 MCS responders^g^ (n=128)	18/45 (40.0)	31/83 (37.3)	N/A	NA	N/A
	VO_2peak_^l^ (ml O_2_/min; n=54)	4.0 (–128.0 to 136.0)	76.3 (5.4 to 147.3)	72.4 (–102.0 to 246.8)	.42	.49
	RPAQ^m^ self-rated PAEE^n^ (kJ/kg/day; n=209)	8.0 (–15.9 to 31.8)	–8.3 (–24.3 to 7.8)	–16.2 (–49.2 to 16.7)	.33	.46
	BREQ-2^o^ RAI^p^ (score –24 to 20)	1.3 (–0.3 to 2.8)	2.6 (1.6 to 3.6)	1.4 (–0.7 to 3.5)	.20	.35
	Weight (kg; n=212)	–0.1 (–1.3 to 1.1)	–1.6 (–2.3 to –0.8)	–1.4 (–3.1 to 0.2)	.09	.21
	Waist circumference (cm; n=212)	–0.9 (–2.2 to 0.5)	–3.1 (–4.0 to –2.2)	–2.3 (–4.1 to –0.4)	.02	.06
**Exploratory secondary outcomes**						
	Sitting time (minutes/day; n=195)	39.5 (–2.4 to 81.4)	–13.0 (–41.8 to 15.8)	–52.5 (–109.6 to 4.6)	NA	N/A
	LPA^q^ time (minutes/day; n=200)	–15.9 (–25.6 to –6.2)	–2.9 (–9.6 to 3.7)	13.0 (–0.5 to 26.4)	NA	N/A
	TPA^r^ level (CPM^s^; n=200)	–12.9 (–41.6 to 15.9)	3.9 (–15.6 to 23.4)	16.8 (–23.1 to 56.6)	NA	N/A
	Steps (n/day; n=200)	–439 (–1100 to 222)	168 (–280 to 616)	607 (–311 to 1525)	NA	N/A
	BMI (kg/m^2^; n=212)	–0.1 (–0.5 to 0.4)	–0.5 (–0.8 to –0.3)	–0.5 (–1.0 to 0.1)	NA	N/A

^a^Data for the StC and IWT groups are presented as least squares mean (95% CI) or n (%) or n/N (%).

^b^StC: standard care.

^c^IWT: interval walking training.

^d^BH: Benjamini-Hochberg.

^e^MVPA: moderate-and-vigorous physical activity.

^f^N/A: not applicable.

^g^MVPA responders were defined as change in MVPA time ≥10 minutes/day; SF-12 PCS responders were defined as change in SF-12 PCS >3.29 [40]; SF-12 MCS responders were defined as change in SF-12 MCS >3.77 [40].

^h^NA: not analyzed.

^i^SF-12: 12-item Short-Form Health Survey.

^j^PCS: Physical Component Summary.

^k^MCS: Mental Component Summary.

^l^VO_2peak_: peak oxygen consumption.

^m^RPAQ: Recent Physical Activity Questionnaire.

^n^PAEE: physical activity energy expenditure.

^o^BREQ-2: Behavioral Regulation in Exercise Questionnaire-2.

^p^RAI: Relative Autonomy Index.

^q^LPA: light-intensity physical activity.

^r^TPA: total physical activity.

^s^CPM: counts per minute.

### Key Secondary Outcomes

From baseline to 12- and 52-week follow-up, SF-12 PCS score (ie, physical HRQoL) increased by a mean of 4.3 (95% CI 3.1 to 5.6) points and 4.2 (95% CI 3.0 to 5.4) points, respectively, in the IWT group and remained unchanged in the StC group. Thus, the SF-12 PCS score increased by a mean of 3.7 (95% CI 1.2 to 6.1) points and 4.3 (95% CI 1.8 to 6.9) points (Benjamini–Hochberg adjusted *P*=.007) more in the IWT group compared with the StC group at 12- and 52-week follow-up, respectively (Multimedia Appendix 6 and Table 2). According to post hoc linear regression analysis, a change in MVPA and IWT duration was not associated with a change in SF-12 PCS over 52 weeks (Multimedia Appendix 8).

Waist circumference decreased by a mean of –3.1 (95% CI –4.0 to –2.2) cm from baseline to 52-week follow-up in the IWT group with no change in the StC group (Table 2). Thus, waist circumference apparently decreased by a mean of –2.3 (95% CI –4.1 to –0.4) cm more in the IWT group compared with the StC group (Benjamini-Hochberg adjusted *P*=.06; Table 2). From baseline to 12-week follow-up, waist circumference decreased by a mean of –2.9 (95% CI –3.7 to –2.0) cm and –3.2 (95% CI –4.5 to –1.9) cm in the IWT and StC group, respectively. No differences were observed between the groups (Multimedia Appendix 6). According to a post hoc linear regression analysis, every 10 minute/day increase in MVPA was associated with a mean decrease of 0.6 (95% CI –1.1 to –0.1) cm in waist circumference (r=–0.21; *P*=.03), while every 10 minute/week increase in IWT duration was associated with a mean decrease of 0.6 (–1.1 to –0.2) cm in waist circumference (r=–0.30; *P*=.008; Multimedia Appendix 8).

No differences in the changes in SF-12 MCS (mental HRQoL), VO_2peak_, RPAQ self-rated PAEE, BREQ-2 RAI, and weight from baseline to 12- and 52-week follow-up, respectively, were observed between the groups (Multimedia Appendix 6 and Table 2).

### Exploratory Secondary Outcomes

Among the exploratory secondary outcomes, no between-group differences were observed from baseline to 12- and 52-week follow-up, except for light-intensity physical activity time and steps that increased from baseline to 12-week follow-up by a mean of 20.1 (95% CI 7.5 to 32.7) minutes/day and 1124 (95% CI 255 to 1992) steps/day, respectively, more in the IWT group compared with the StC group (Multimedia Appendix 6 and Table 2).

### Subgroup Analyses

**Figure 3:**
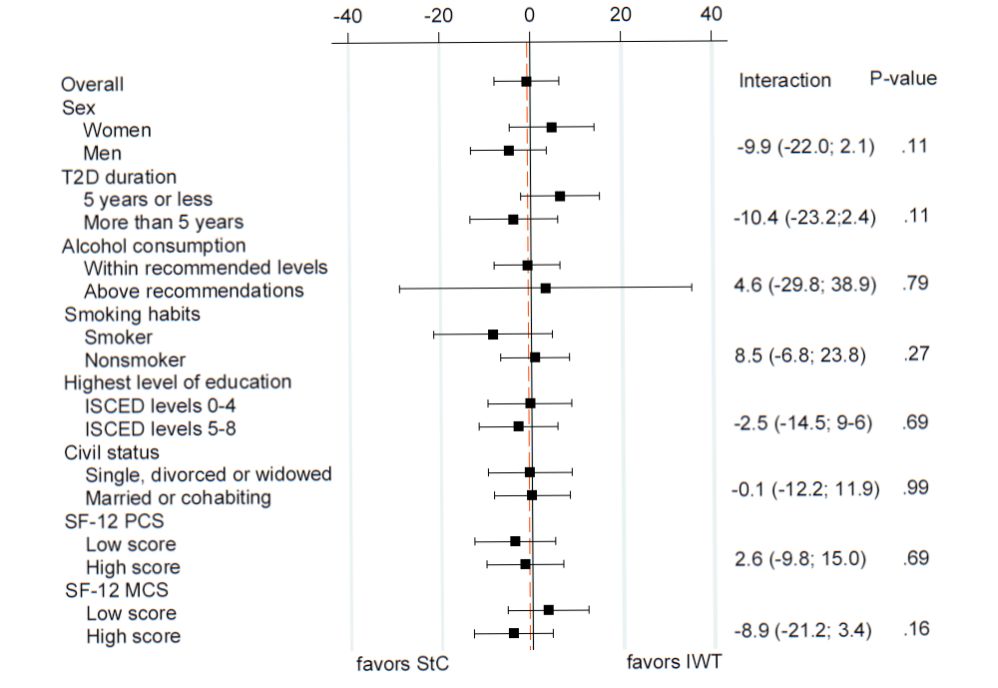
Forest plot of overall and subgroup effects of IWT vs. StC on change in MVPA time (min/day) after 52 weeks. Subgroup effects include sex (men/women), type 2 diabetes duration (≤5 years/>5 years), alcohol consumption (within the recommended levels/above recommendations), smoking habits (smoker/nonsmoker), highest level of education (ISCED-2011 levels 0-4/ISCED-2011 levels 5-8), civil status (single, divorced or widowed/married or cohabiting), SF-12 PCS (high/low), and SF-12 MCS (high/low), assessed at baseline. ISCED: International Standard Classification of Education; IWT: interval walking training; MCS: Mental Component Summary; PCS: Physical Component Summary; SF-12: 12-item Short-Form Health Survey; StC: standard care; T2D: type 2 diabetes.

From baseline to 52-week follow-up, there were no differences between the IWT_only_ and IWT_support_ groups in the changes in primary, key secondary, and exploratory secondary outcomes (Multimedia Appendix 9). Moreover, no subgroup effects of IWT versus StC on the change in MVPA time were observed (*P*>.1 for interaction; Figure 3), nor were there any differences in mean IWT duration over 52 weeks among the subgroups in the IWT group (Multimedia Appendix 10).

### Harms

No adverse events or harms were reported to the health professionals.

## Discussion

The main finding of this study was that InterWalk app–based IWT did not increase objectively measured MVPA time over 52 weeks compared with standard care among individuals with T2D referred to municipality-based lifestyle programs. While InterWalk app–based IWT resulted in improvements in physical HRQoL and nonsignificant reduction in waist circumference compared with StC, no effects were observed in mental HRQoL, physical fitness, or weight. A key objective of this study was to support individuals with T2D in maintaining PA levels after a 12-week municipality-based exercise program with minimal direct support from health professionals. In general, IWT adherence was low across 52 weeks, largely explaining the lacking effect on MVPA time. During the 12-week exercise program, IWT adherence was remarkably higher and MVPA time increased by >10% from baseline (by 3.6 minutes/day from 30.2 minutes at baseline), indicating that InterWalk app–based IWT potentially contributed to increased MVPA time. The discrepancy of the IWT adherence observed in this and previous IWT studies among individuals with T2D indicates that InterWalk app–based IWT may not—in the present circumstances—be feasible for maintaining PA level after a municipality-based exercise program [19,24].

In line with previous studies, adherence to the exercise intervention decreased over time [12] when supervision was subtracted [15]. In contrast to our observations, previous reports observed increases in objectively measured PA (such as MVPA, steps, and moderate-intensity walking time) following digitally supported PA interventions [41-44]. This discrepancy may partly be explained by shorter intervention periods of the previous studies [41-44]. Indeed, intermediate assessment after only 12 weeks in this study involved increases in MVPA time, although this effect may be driven by the additional direct supervision integral to the exercise program. As such, discrepancies may further be explained by differences in other intervention features, such as health professional consultations or group sessions in previous studies, and features of the applied digital solutions, including web-based solutions or smartphone apps [41-44]. These digital solutions were designed to support PA adoption, for example, through goal setting and self-management [41-44], whereas the InterWalk app was designed as a training device specifically to deliver individually tailored IWT [20]. However, in contrast to previous findings [41-44], providing motivational interviews with individual goal setting in addition to the InterWalk app did not influence intervention adherence or outcomes over 52 weeks.

In line with this study, previous studies have observed decreased waist circumference [10,11,44] and improved self-reported physical health and quality of life [43,45] after a PA intervention. We observed that decreased waist circumference was maintained 40 weeks after the 12-week exercise program when other intervention features were discontinued, and participants only had access to the InterWalk app. This observation is supported by similar results in the eCoFit trial over just 10 weeks following a smartphone-supported PA intervention, featuring workout circuits using the outdoor environment, social support, goal setting, etc. [44]. Post hoc analyses suggested an inverse dose-response relationship of both IWT and MVPA with waist circumference. Accordingly, Ross and colleagues [46] observed that 24 weeks of supervised high-volume exercise decreased waist circumference among individuals with obesity. We observed similar decreases in waist circumference across 52 weeks with access to the InterWalk app and markedly less direct supervision. These results may be of clinical relevance as 1-cm decreases in waist circumference is associated with 4% reductions in visceral fat mass [47]. Thus, the observed 3-cm decrease in waist circumference is likely associated with substantial reductions in visceral fat mass, which potentially lead to considerable improvements in glycemic control and decreased low-grade inflammation [19,47,48]. Likewise, improved physical HRQoL was maintained 40 weeks after the exercise program when participants only had access to the InterWalk app. This is supported by similar results in a previous study, where physical HRQoL was preserved following a 12-month intensive lifestyle intervention [45]. Post hoc analyses suggested that the improved physical HRQoL across 52 weeks in this study was independent of changes in MVPA and IWT duration, indicating that this effect was driven by other mechanisms than PA behavior change. This is supported by a previous study observing no effects of a 1-year behavior change intervention on objectively measured PA, although higher levels of self-reported physical functioning compared with standard care were reported [49]. In this study, possible mechanisms may be related to the safe and convenient nature of IWT, potentially affecting participants’ perception of their physical health, or to the continued access to app-supported PA. However, when participants in a previous study were provided access to a digital monitoring and feedback solution, the effects of self-management support on self-reported physical health were inhibited [41].

Limitations of this study include the high loss to follow-up corresponding to 39% at 52 weeks and the low adherence to the intervention. As indicated by the high adherence levels in previous IWT studies among individuals with T2D [19,24], these low adherence levels may result from the pragmatic approach to the study design. Accordingly, the applied intervention has previously showed high level of efficacy in explanatory trials including rather homogenous samples and increased standardization and control [19,24]. With our study design, we did not identify a feasible solution for effective implementation of this efficacious intervention in municipality-based health care of individuals with T2D. One limitation of our approach includes the lack of detailed information on the adherence to and delivery of the co-interventions during the municipality-based rehabilitation programs from baseline to 12-week follow-up. Besides, the lack of objective monitoring of the adherence to the follow-up intervention and the single features of the InterWalk app preclude us from drawing strong conclusions of the effectiveness of the InterWalk app per se*.* Moreover, according to descriptive post hoc analyses, baseline demographic and clinical characteristics among attenders and nonattenders at 52-week follow-up did not seem to be different (Multimedia Appendix 11), and thus did not appear to explain the high loss to follow-up. Further, the sample representativeness was limited. First, individuals of low socioeconomic status are less likely to be referred to municipality-based lifestyle programs [50]. Second, choosing to participate in an exercise program indicates a will to change PA behavior, whereas rejecting participation may not. Third, as Danish language was used during assessment (eg, questionnaires) and exercise programs, non-Danish speaking individuals were precluded from participation. Altogether, this sample may underrepresent the most vulnerable individuals with T2D. Further, our sample was more physically active at baseline than the general US population [51] and individuals with T2D in a previous study [52]. This indicates potential ceiling effect of MVPA at baseline and thus selection bias by exclusion of less physically active individuals from this sample. This stresses the need for development of efficacious, contemporary approaches to support PA adherence and adoption with the potential to increase PA levels among the wide population of individuals with T2D. Finally, we experienced technical malfunction of the InterWalk app related to a major restructuring of the iOS, which may have affected data upload and collection during that period. However, this was not reflected in the sensitivity analysis excluding participants undergoing intervention during this period.

In conclusion, among individuals with T2D referred to municipality-based lifestyle programs, randomization to InterWalk app–based IWT did not increase objectively measured MVPA time over 52 weeks compared with standard care, although an improvement in physical HRQoL was observed. Moreover, in this municipality-based setting, adherence to the intervention was low even when additional motivational support was provided. Further research is needed to identify optimal implementation of digital support for PA adherence and adoption among individuals with T2D.

## Appendix

Multimedia Appendix 1 Statistical analysis plan.

Multimedia Appendix 2 Accelerometer wear time and nonwear time at baseline, 12-week, and 52-week follow-up.

Multimedia Appendix 3 Graph of mean (SD) intensities (per minute) of all registered IWT sessions in the IWT group (ie, IWTonly + IWTsupport).

Multimedia Appendix 4 Adherence to the intervention in the IWT combined (IWTsupport + IWTonly), IWTonly and IWTsupport groups.

Multimedia Appendix 5 Overview of adherence to the follow-up motivational interviews for the IWT support group.

Multimedia Appendix 6 Intention-to-treat analysis of changes from baseline to 12-week intermediate assessment between the IWT group and the StC group.

Multimedia Appendix 7 Sensitivity analyses of changes in MVPA time from baseline to 52-week follow-up between the IWT group and the StC group: (1) Per-protocol analysis; (2) Analysis with non-responder (baseline observation carried forward; BOCF) imputation; (3) Analysis with the subsample of participants whose intervention period did not overlap time of technological malfunction of the InterWalk application; and (4) Analysis with daytime criteria for accelerometer data (ie, ≥3 days of ≥14 h of daytime measurement (6 am to 22 pm)). (5) Sensitivity analysis of changes in forced walking (ie, ≥3000 CPM) time from baseline to 52-week follow-up between the IWT group and the StC group.

Multimedia Appendix 8 Post hoc linear regression analysis of the dose response association over 52 weeks between (a) change in MVPA (min/day) and change in SF-12 PCS score; (b) IWT duration (min/week) and change in SF-12 PCS score; (c) change in MVPA (min/day) and change in waist circumference (cm); and (d) IWT duration (min/week) and change in waist circumference (cm).

Multimedia Appendix 9 Intention-to-treat analysis of changes from baseline to 52-week follow-up between the IWTonly group and the IWTsupport group.

Multimedia Appendix 10 Bar chart of median (25th; 75th percentile) IWT duration (min/week) over 52 weeks among IWT subgroups of sex (men/women), type 2 diabetes duration (≤5 years/>5 years), alcohol consumption (within the recommended levels/above recommendations), smoking habits (smoker/nonsmoker), highest level of education (ISCED-2011 levels 0-4/ISCED-2011 levels 5-8), civil status (single, divorced, or widowed/married or cohabiting), SF-12 PCS (high/low), and SF-12 MCS (high/low), assessed at baseline.

Multimedia Appendix 11 Baseline demographic and clinical characteristics among attenders and nonattenders at 52-week follow-up.

Multimedia Appendix 12 CONSORT-eHEALTH checklist (V 1.6.1).

## Abbreviations

BREQ-2: Behavioral Regulation in Exercise Questionnaire-2
CPM: counts per minute
HRQoL: health-related quality of life
ISCED: International Standard Classification of Education
IWT: interval walking training
LPA: light-intensity physical activity
LS: least square
MCS: Mental Component Summary
MVPA: moderate-and-vigorous physical activity
PA: physical activity
PAEE: physical activity energy expenditure
PCS: Physical Component Summary
RAI: Relative Autonomy Index
RPAQ: Recent Physical Activity Questionnaire
SF-12: 12-item Short-Form Health Survey
StC: standard care
T2D: type 2 diabetees
TPA: total physical activity
VO_2_peak: peak oxygen consumption
