# Supplementary material for: The Effects of a Lifestyle Intervention Supported by the InterWalk Smartphone App on Increasing Physical Activity Among Persons With Type 2 Diabetes: Parallel-Group, Randomized Trial
Source: JMIR Mhealth Uhealth. 2022 Sep 28;10(9):e30602. doi: 10.2196/30602 (PMC9557767; doi:10.2196/30602)
Supplement: Multimedia Appendix 1 [file mhealth_v10i9e30602_app1.pdf]

STATISTICAL ANALYSIS PLAN (SAP)

Long-term Effectiveness of Smartphone App-Delivered Interval Walking Training on Physical Activity Among Individuals with Type 2 Diabetes: SAP for the parallel-group, assessor-blinded, randomized InterWalk trial

Trial registration number: NCT02341690

SAP version (1.1) (December 9, 2020)

Protocol version: doi:10.1136/bmjopen-2016-014036

SAP authors:

Ida Kær Thorsen, Centre for Physical Activity Research (CFAS), Rigshospitalet, University of Copenhagen, Denmark

Yanxiang Yang, Technische Universität München, Munich, Germany, and Centre for Physical Activity Research (CFAS), Rigshospitalet, University of Copenhagen, Denmark

Mathias Ried-Larsen, Centre for Physical Activity Research (CFAS), Rigshospitalet, University of Copenhagen, Denmark

Collaborators:

The Danish Center for Strategic Research in Type 2 Diabetes, Denmark.

Center for Diabetes, Municipality of Copenhagen, Denmark.

Statistical advisor:

Robin Christensen, Professor of Biostatistics and Clinical Epidemiology; Musculoskeletal Statistics Unit, the Parker Institute, Bispebjerg and Frederiksberg Hospital, University of Copenhagen, Copenhagen & Research Unit of Rheumatology, Department of Clinical Research, University of Southern Denmark, Odense University Hospital, Denmark.

Statistical analyst:

Ida Kær Thorsen, Centre for Physical Activity Research (CFAS), Rigshospitalet, University of Copenhagen, Denmark

BACKGROUND AND RATIONALE

Physical activity (PA) is considered a cornerstone in the prevention and management of type 2 diabetes (T2D) [1]. Supervised PA is effective in improving glycemic control, whereas advice-based PA is not [2]. With the increasing prevalence of T2D [3], there is an acute need for easily accessible alternatives for long-term remote PA support.

Inspired by previous studies reporting significant health benefits of Interval Walking Training (IWT) [4-6], the InterWalk application was developed in Denmark to deliver individually tailored IWT to individuals with T2D [7]. The InterWalk application guides and paces the user through an individually chosen number of repeated cycles of 3 minutes slow walking and 3 minutes fast walking.

The primary hypothesis of the present study is that InterWalk app-delivered IWT is superior to standard care in increasing moderate-to-vigorous PA (MVPA) time after 52 weeks.

OBJECTIVES

- The primary objective of this study will be to investigate the effectiveness of InterWalk app-delivered IWT compared with standard care in increasing objectively measured MVPA from baseline to week 52 in individuals with T2D.
- Key secondary objectives will be to investigate to what extend IWT, compared with standard care, induce changes in physical and mental health-related quality of life (HR-QoL), physical fitness (VO_{2peak}), self-reported physical activity energy expenditure (PAEE), motivation for physical activity and behavior change (BREQ-2), body weight, and waist circumference from baseline to week 52.
- Exploratory secondary objectives include investigations of the effects of the intervention on objectively measured sitting time, light-intensity physical activity (LPA) time, total physical activity (TPA) level, steps, and body mass index (BMI) from baseline to week 52.

STUDY METHODS

TRIAL DESIGN

The study was conducted as a parallel-group, assessor-blinded, randomized controlled trial with 52 weeks intervention and follow-up. All groups began with 12 weeks supervised exercise program. The materials and methods including the trial design is described in detail in the published protocol [8] and briefly summarized below.

To investigate the primary objective, we used a parallel group design allocation ratio of 2:1. Accordingly, the participants were randomly allocated into; (1) structured InterWalk app-delivered IWT (2/3; IWT group), and (2) standard care (1/3; StC group (control)). Following the initial 12-week supervised exercise program, the participants in the IWT group were further randomly allocated 1:1 into (A) IWT, no additional support (IWT_{only} group), and (B) IWT, with additional support ($IWT_{support}$ group). I.e. participants in the IWT_{only} group and $IWT_{support}$ group underwent similar interventions during the 12-week exercise program, and allocation to either of these were concealed until after intermediate assessment at week 12.

The InterWalk app-delivered IWT (30-60 min per sessions, 3 times per week for 12 weeks) was group-based and two of three sessions were supervised during the 12-week exercise program. An IWT bout consisted of repeated cycles of 3 minutes of slow and 3 minutes of fast walking [9]. The StC group received standard care, including a standard supervised group-based exercise program (twice per week for 12 weeks). Exercise programs in all three arms were delivered by health professionals at the municipality or hospital during the 12-week exercise program.

All participants were encouraged to continue exercising after the 12-week exercise program. Participants in the StC group and IWT_{only} group did not receive any support from the health professionals at the health promotion center or hospital after the 12-week exercise program. Participants in the $IWT_{support}$ group received support, including individual motivational interviews with individual goal setting (week 16, 20, 28 and 40), SMS support (once per week) and IWT with ambassadors (once per week).

During the 12-week exercise program period, all participants were offered simultaneous additional lifestyle programs, including disease-related education, diet counseling, alcohol counseling and smoking cessation, as a part of the standard care at the municipality or hospital.

RANDOMIZATION, BLINDING AND CONCEALMENT

Participants were randomized using random permuted blocks, stratified by sex (2 levels) and center (6 levels) to ensure balance and equal representation in all groups. The allocation sequence was generated through a standardized computer program and stored on a password-protected computer by an independent researcher (RØN) who was not involved in any study procedures. Following the baseline tests, which were conducted by the health professionals at the health promotion centers, the independent researcher was contacted and performed the allocation (to StC or IWT_{only} or $IWT_{support}$). The respective group allocation for the initial 12 weeks (StC or IWT) was subsequently returned to the health professional who informed the participant about the allocation. Information about IWT_{only}

or $IWT_{support}$ was not disclosed at this stage. Following the 12-week intermediate assessment, the indpendent researcher was contacted and returned the allocation into IWT_{only} or $IWT_{support}$ to the health professional who informed the participant about the allocation. Only the scientific staff were blinded to the allocation as the health professionals carried through the intervention.

SAMPLE SIZE

Sample size considerations are described in the published protocol [8]. In brief, the minimal important difference (MID) was considered to be 10 minutes of MVPA per day. We assumed the standard deviation (SD) of the change in MVPA time from baseline to 52-week follow-up to be between 1.2 and 2.3 times the effect size. Therefore, SD twice the MID (20 minutes of MVPA per day) was applied in the sample size calculations. Bonferroni adjustment due to multiple comparisons in the three-group trial were considered in the power calculation. A total of 190 participants were needed to obtain a statistical power (1-β) of 80% with an α level of 0.017 (two-tailed) using an unpaired t-test. Allowing for 30% attrition, 272 patients (91 in the StC group and 181 in experimental group) should be recruited. The intervention settings enabled recruitment until December 15, 2016, and thus the sample size would include 272 participants or truncated at the number of participants reached at the end of recruitment period—whichever was reached first. On December 15, 2016, 213 participants had been included and they constitute the final analysis population.

FRAMEWORK

For the primary objective, a superiority hypothesis testing framework will be employed to investigate the effectiveness of InterWalk app-delivered IWT in increasing MVPA time after 52 weeks compared with standard care.

TIMING OF FINAL ANALYSIS

Final analysis will be conducted following 52-week follow-up of the last participant and completion of the statistical analysis plan.

TIMING OF OUTCOME ASSESSMENTS

Timing of outcome assessments is described in Table 3 of the published protocol [8]. Objectively measured MVPA time was assessed at baseline, 12-week, and 52-week follow-up. The 52-week follow-up was finalized 52 weeks after recruitment of the last participant, i.e. in December 2017.

STATISTICAL PRINCIPLES

CONFIDENCE INTERVALS AND P VALUES

All 95% confidence intervals (95%CI) and *P*-values are two-sided. Since more than one comparison is made, the chance of falsely detecting a nonexistent effect increases (i.e. due to multiple comparisons), and thus *ad hoc* adjustments will be made to account for this in the interpretation. As such, the analyses of the key secondary outcomes will be performed in sequence (based on *P*-values) until one of the analyses fails to show the statistically significant difference, or until all analyses have been completed at a statistical significance level of 0.05. Accordingly, key secondary outcome analyses will be controlled for the false discovery rate (FDR) using the Benjamini-Hochberg step-up procedure [10]. As such, p-values for the *m* tests will be computed and ordered lowest to highest (i.e. $p_{(1)} \leq p_{(2)} \leq \ldots \leq p_{(m)}$), the largest *k* for which $\frac{m}{k} \cdot p_{(k)} \leq \alpha$ will be identified, and the null hypotheses corresponding to $p_{(1)} \leq p_{(2)} \leq \ldots \leq p_{(k)}$ will be rejected.

ADHERENCE AND PROTOCOL DEVIATIONS

IWT sessions (incl. frequency, duration and intensity) using the InterWalk app was electronically logged in the app and uploaded to a server across the 52 weeks of participation in the trial. For the IWT_{only}, $IWT_{support}$, and IWT (IWT_{only} + $IWT_{support}$) groups, frequency, duration and intensity of exercise across 52 weeks will be presented in Supplementary Table 1. Mean intensity (per 30 seconds) of all registered IWT sessions in the IWT group (i.e. IWT_{only} + $IWT_{support}$); the IWT_{only} group; and the $IWT_{support}$ group, will be presented in Supplementary Figure 1. Moreover, for the $IWT_{support}$ group, self-reported adherence to IWT will be calculated based on SMS-track data (categorically presented as numbers and proportions) and presented in Supplementary Table 1.

ANALYSIS POPULATIONS

The primary analyses will be based on the full analysis set using an intention to treat (ITT) approach. The ITT principle asserts the effect of a treatment policy (i.e. the planned treatment regimen) rather than the actual treatment given (i.e. independent of adherence) [11]. Accordingly, participants allocated to a treatment group will be followed up, assessed and analyzed as members of that group, irrespective of adherence to the planned course of treatment (i.e., independent of intercurrent events such as withdrawals and cross-over phenomena). Missing data will be handled indirectly and statistically modeled using repeated-measures linear mixed models (see below) [12].

In addition to the ITT approach, data will be analyzed per protocol as a sensitivity analysis. We define the per-protocol population in all groups as persons completing the assessment of the primary outcome measure at baseline, 12-week and 52-week follow-up (i.e. complete cases). For the intervention group participants should have completed ≥70% of the prescribed exercise volume during the 52-week intervention. For the StC group we define the per protocol population as participants with no registered IWT sessions in the InterWalk app.

TRIAL POPULATION

ELIGIBILITY

Eligibility for participation in the trial was based on following criteria. Inclusion criteria were T2D diagnosis, >18 years of age, and referral to a health promotion center or hospital in the participating municipality by their general practitioner. Exclusion criteria were medical contraindications to exercise, e.g. chronic complications in the musculoskeletal system, painful osteoarthritis or heart conditions; declining to participate in a PA program at the health promotion center or hospital; current participation in other intervention studies; or insufficient language skills (Danish). Potential participants were screened through medical records and at a screening interview with a health professional at the health promotion center or hospital.

RECRUITMENT

All individuals with T2D referred to the health promotion center or hospital in the participating municipalities were screened for eligibility. Municipalities included Copenhagen (four health promotion centers: Amager, Vanløse, Østerbro and Vesterbro), Guldborgsund (health promotion center) and Bornholm (hospital). Recruitment began in January 2015 and ended in December 2016.

WITHDRAWAL/FOLLOW-UP

The participant flow will be presented in Figure 1. A total of 60 participants did not provide primary outcome data at 12-week intermediate assessment, resulting in a loss to follow-up of 28%. Further 24 participants (from baseline, a total of 84 participants) did not provide primary outcome data at 52-week follow-up, resulting in a loss to follow-up of 39%. Loss to follow-up will be presented in a participant flow diagram in Figure 1.

BASELINE PARTICIPANT CHARACTERISTICS

Baseline characteristics that will be summarized include sex; age; T2D duration; alcohol consumption; smoking habits; highest level of education; civil status; height; MVPA time; 12-item Short-Form Health Survey (SF-12) Physical Component Summary (PCS) and Mental Component Summary (MCS); VO_{2peak}; Resent Physical Activity Questionnaire (RPAQ) self-rated PAEE; Behavioural regulation in exercise questionnaire-2 (BREQ-2) relative autonomy index (RAI); weight; waist circumference; sitting time; LPA time; TPA level; steps; and BMI.

ANALYSIS

OUTCOME DEFINITIONS

Primary outcome: Change in MVPA time (min/day) from baseline to 52-week follow-up. PA level was measured using accelerometers (Axivity AX3, Newcastle, UK). Participants wore two accelerometers (thigh and back) for 7-10 consecutive days. MVPA time was defined according to Freedson's cut point, ≥1952 counts per minute (CPM) [13], using the vertical axis of the accelerometer placed on the back. Participants were included in the analyses of objectively measured PA if they had ≥3 days of ≥22 h of measurement (i.e. allowing for 2 h non-wear time).

Key secondary outcome measures:

1. Change in the SF-12 PCS (score 0-100) from baseline to 52-week follow-up [14]. PCS was calculated based on four subscales, including Physical Functioning (PF), Role Physical (RP), Bodily Pain (BP), and General Health (GH) [14].
2. Change in the SF-12 MCS (score 0-100) from baseline to 52-week follow-up [14]. MCS was calculated based on four scales, including Vitality (VT), Social Functioning (SF), Role Emotional (RE), and Mental Health (MH) [14].
3. Changes in VO_{2peak} (ml O_2/min) from baseline to 52-week follow-up. VO_{2peak} was estimated using the standardized walking test in the InterWalk app [9].
4. Change in RPAQ self-rated PAEE (kJ/day) from baseline to 52-week follow-up.
5. Change in BREQ-2 RAI (score −24-20) from baseline to 52-week follow-up.
6. Change in weight (kg) from baseline to 52-week follow-up.
7. Change in waist circumference (cm) from baseline to 52-week follow-up.

Exploratory secondary outcomes include:

- Change in sitting time (min/day) from baseline to 52-week follow-up.

- Change in LPA time (min/day) from baseline to 52-week follow-up. LPA time was defined according to Freedson's cut point, ≥100 CPM and ≤1951 CPM [13], using the vertical axis of the accelerometer placed on the back.
- Change in TPA level (CPM) from baseline to 52-week follow-up measured using the vertical axis of the accelerometer placed on the back.
- Change in Steps (n/day) from baseline to 52-week follow-up measured using the vertical axis of the accelerometer placed on the back.
- Change in BMI (kg/m^2) from baseline to 52-week follow-up.

Protocol changes were implemented after the trial registration.

- Change in endurance in the lower extremities (30-sec sit-to-stand test) was excluded from the test protocol due to unexpected measurement time restrictions.
- Change in body temperature measured using an ear thermometer was excluded from the test protocol based on feedback from the participating health promotion centers and hospital.

ANALYSIS METHODS

Baseline characteristics: Characteristics of the study population at baseline will be reported in Table 1, using descriptive statistics, i.e. mean and SD (median and IQR) will be reported for continuous variables, and numbers and proportions will be reported for categorical variables. Data will tentatively be checked for missing values, outliers and normality.

Primary analyses: The primary analyses will be based on the ITT population, i.e. using continuous data (change from baseline as the dependent variable) as observed, including all randomized participants with available data at baseline. Missing data will be handled indirectly and statistically modelled using repeated-measures linear mixed models. Contrasts between groups (IWT vs. StC) after 52 weeks will be estimated based on repeated-measures, mixed linear models, with the baseline value of the relevant variable as a covariate, including fixed effect factors for group#1 (2 levels: IWT/StC), group#2 (3 levels: IWT_{only}/$IWT_{support}$/StC), time (3 levels: baseline/12-week/52-week), and group×time interaction); and random effects (subject ID). We will also adjust for the stratifying factors sex (2 levels: male/female), 'clinical center' (6 levels: Amager/Vanløse/Østerbro/ Vesterbro/Guldborgsund/Bornholm).

The model will be checked according to the following assumptions 1) linearity, 2) normality of residuals, 3) homogeneity of residuals variance, and 4) independence of residuals error. The

change in MVPA time from baseline to 52-week follow-up will be reported in Table 2 as Least squares means with standard errors for each group (IWT and StC group, respectively), and the difference between them will be presented with 95% CI's.

Secondary analyses: Categorical outcomes for dichotomous endpoints (including responder status and harms) will be analyzed with logistic regression with binary outcomes assessed after 52 weeks (i.e. dependent variable), with the same fixed effect factors and covariates as the respective analysis for continuous outcomes. Since Odds Ratios (ORs) for outcomes of common incidence either over- or under-estimate the corresponding risk estimate, we will convert all the calculated OR values and 95% confidence intervals into approximate Risk Ratios (RR) in the text.

Other secondary analyses:

- Changes from baseline to 12-week intermediate assessment will be reported separately in Supplementary Table 2.
- The primary and secondary outcome analyses will be repeated without combining the two IWT groups, i.e. with three groups (IWT_{only} vs. $IWT_{support}$ vs. StC) and three time points (baseline, 12-week and 52-week follow-up). Changes from baseline to 52-week follow-up will be reported in Supplementary Table 3.

Sensitivity analyses:

The following five sensitivity analyses of changes in MVPA time from baseline to 52-week follow-up between the IWT group and the StC group will be reported in Supplementary Table 4:

1. Per-protocol analyses will be conducted to investigate robustness of the primary analysis. Per-protocol criteria are described in the *Analysis populations* subsection.
2. Sensitivity regarding imputation of missing observations will be tested by conducting the analysis on the ITT population with a conservative single-imputation non-responder imputation technique (i.e. missing data replaced with the baseline observation carried forward [BOCF]).
3. Sensitivity regarding the potential influence of technical malfunctions of the InterWalk app in relation to a major restructuring of the IOS (version 9; available during the period September 16–October 21, 2016) will be tested by repeating the primary analysis with the subsample of participants whose intervention period did not overlap the period of technological malfunction of the InterWalk application.

4. Sensitivity regarding the measurement of MVPA will be tested by repeating the primary analysis with an alternative cut-off at 3000 CPM (i.e. corresponding to forced walking).
5. Sensitivity regarding the measurement of MVPA will be tested by repeating the primary analysis with daytime criteria, i.e. ≥3 days of ≥14 h of daytime measurement (6 am to 22 pm; i.e. allowing for 2 h of non-wear time during the specified period).

Subgroup analyses:
Subgroup analyses will be conducted to investigate whether there is a potential interaction between groups and various patient characteristics on change in MVPA time after 52 weeks among subgroups of:

- Sex (men/women)
- T2D duration (≤5 years/>5 years)
- Alcohol consumption (within the recommended levels/above recommendations)
- Smoking habits (smoker/nonsmoker)
- Highest level of education (short/long education)
- Civil status (single, divorced or widowed/married or cohabiting)
- Baseline PCS level (high/low)*
- Baseline MCS level (high/low)*

*PCS and MCS will divided into high and low using median split.

Subgroup analyses of the IWT group will be conducted to investigate potential differences in total duration of IWT sessions over 52 weeks among above-mentioned subgroups.

MISSING DATA
The patterns of missing data will be investigated prior to the formalized statistical analyses. Repeated Measurements Using Mixed Models: Our primary analyses will be based on the ITT population, including all randomized participants with available data at baseline. Missing data will be handled indirectly and statistically modeled using repeated-measures linear mixed models (see below). These models will be valid if data are 'Missing at Random' (MAR): "Any systematic difference between the missing values and the observed values can be explained by differences in observed data" [12].

HARMS

The study was expected to result in limited risks, adverse effects or discomfort to the participants. No invasive methods were used. The IWT caused some degree of breathlessness in the fast walking interval periods. The anthropometric measurements, questionnaires, and motivational initiatives were not associated with any known risk or other discomforts. Adverse events will be reported (numbers and proportions).

STATISTICAL SOFTWARE

All statistical analyses will be performed using StataIC 13 (StataCorp, Tx, USA).

PROGRAMMING PLAN

The program code for the analysis of the primary outcome in Stata is;

****

mixed $MVPA_{change}$ *group1##time* $MVPA_{baseline}$ *group2 sex center* || *ID*:,

****

where $MVPA_{change}$ is change in MVPA time in min/day from baseline to 52-week follow-up; *group1* is group allocation with two groups (2 levels; IWT or StC); *time* is the time variable (3 levels; baseline, 12-week and 52-week); *group2* is group allocation with three groups (3 levels; IWT_{only} or $IWT_{support}$ or StC); *sex* is biological sex (2 levels; man or woman); *center* is the test and intervention center (6 levels; health promotion centers in Amager, Vanløse, Østerbro, Vesterbro, and Guldborgsund; and hospital in Bornholm); and *ID* is subject ID.

ANTICIPATED OUTLINE OF THE MANUSCRIPT

Figure 1: Participant flow chart.

Figure 2: Least squares means (95% confidence interval) of MVPA time (min/day) at baseline, 12-week and 52-week follow-up for the IWT and StC group.

Figure 3: Forest plot of overall and subgroup effects of IWT vs. StC on change in MVPA time (min/day) after 52 weeks. Subgroup effects include sex (men/women), T2D duration (≤5 years/>5 years), alcohol consumption (within the recommended levels/above recommendations), smoking habits (smoker/nonsmoker), highest level of education (short/long education), civil status (single, divorced or widowed/married or cohabiting), PCS (high/low), and MCS (high/low), assessed at baseline.

Supplementary Figure 1: Graph of mean (SD) intensities (per 30 seconds) of all registered IWT sessions in (A) the IWT group (i.e. IWT_{only} + $IWT_{support}$); (B) the IWT_{only} group; and (C) the $IWT_{support}$ group.

Supplementary Figure 2: Bar chart of mean (95% CI) IWT duration over 52 weeks among IWT subgroups of sex (men/women), T2D duration (≤5 years/>5 years), alcohol consumption (within the recommended levels/above recommendations), smoking habits (smoker/nonsmoker), highest level of education (short/long education), civil status (single, divorced or widowed/married or cohabiting), PCS (high/low), and MCS (high/low), assessed at baseline.

Table 1: Demographic and clinical characteristics of participants at baseline

	IWT group	StC group	Total
Sex:			
Male, n (%)			
Female, n (%)			
Age (years)			
T2D duration:			
Median T2D duration (years)			
≤5 years, n (%)			
>5 years, n (%)			
Alcohol consumption:			
Within the recommended levels, n (%)			
Above recommendations, n (%)			
Smoking habits:			
Smoker, n (%)			
Nonsmoker, n (%)			
Highest level of education:			
Short education, n (%)			
Long education, n (%)			
Civil status:			
Single, divorced or widowed, n (%)			
Married or cohabiting, n (%)			
Height (cm)			
Primary outcome			
MVPA time (min/day)			
Key secondary outcomes			
SF-12 Physical Component Summary (PCS) (score 0-100)			
SF-12 Mental Component Summary (MCS) (score 0-100)			
VO_{2peak} (ml O_2/min)			
RPAQ self-rated PAEE (kJ/day)			
BREQ-2 RAI (score −24-20)			
Weight (kg)			
Waist circumference (cm)			
Key secondary outcomes with incomplete assessment			
Endurance in the lower extremeties (n/30 sec)	n/a	n/a	n/a
Body temperature (°C)	n/a	n/a	n/a
Exploratory secondary outcomes			
Sitting time (min/day)			
LPA time (min/day)			
TPA level (CPM)			
Steps (n/day)			
BMI (kg/m^2)			

Data are means (standard deviations), medians (interquartile ranges), or numbers (proportion).

Abbreviations: IWT, interval walking training; StC, Standard care; T2D, type 2 diabetes; BMI, body mass index; MVPA, moderate-to-vigorous physical activity; SF-12, the Short-Form health Survey; PAEE, physical activity energy expenditure; BREQ-2, Behavioural regulation in exercise questionnaire-2; RAI, relative autonomy index; VO_{2peak}, peak oxygen consumption; n/a, not applicable; LPA, light physical activity; TPA, total physical activity; CPM, counts per minute.

Variables marked with red were not measured.

Table 2: Intention-to-treat analyses of changes from baseline to 52 week follow-up between the IWT group and the StC group*

	IWT group	StC group	Between-group difference	
	LS Mean ± SE	LS Mean ± SE	Difference between means (95% CI)	*P*-value
Primary outcome				
MVPA time (min/day)				
#MVPA responders, n (%)				*N.A.*
Key secondary outcomes				
SF-12 Physical Component Summary (PCS) (score 0-100)				
#SF-12 PCS responders, n (%)				*N.A.*
SF-12 Mental Component Summary (MCS) (score 0-100)				
#SF-12 MCS responders, n (%)				*N.A.*
VO_{2peak} (ml O_2/min)				
RPAQ self-rated PAEE (kJ/day)				
BREQ-2 RAI (score −24-20)				
Weight (kg)				
Waist circumference (cm)				
Key secondary outcomes with incomplete assessment				
Endurance in the lower extremeties (n/30 sec)	n/a	n/a	n/a	*N.A.*
Body temperature (°C)	n/a	n/a	n/a	*N.A.*
Exploratory secondary outcomes				
Sitting time (min/day)				*N.A.*
LPA time (min/day)				*N.A.*
TPA level (CPM)				*N.A.*
Steps (n/day)				*N.A.*
BMI (kg/m^2)				*N.A.*

Data are LS Means (standard errors) and Difference between means (95% CI's). #will be reported as Odds Ratios (95% CI's).

*Missing data for the ITT population will be handled using repeated-measures mixed linear models for continuous outcomes; and 5 sets of multiple imputations will be applied for the responder indices after 52 weeks.

MVPA responders are defined as change in MVPA time ≥10 min/day; SF-12 PCS responders are defined as change in SF-12 PCS >3.29 [15]; SF-12 MCS responders are definded as change in SF-12 MCS >3.77 [15].

Abbreviations: IWT, interval walking training; StC, Standard care; LS Means, Least Squares Means; CI, Confidence Interval; MVPA, moderate-to-vigorous physical activity; N.A., not analyzed; SF-12, the Short-Form health Survey; RPAQ, Resent Physical Activity Questionnaire; PAEE, physical activity energy expenditure; BREQ-2, Behavioural regulation in exercise questionnaire-2; RAI, relative autonomy index; VO_{2peak}, peak oxygen consumption; n/a, not applicable; LPA, light physical activity; TPA, total physical activity; CPM, counts per minute; BMI, body mass index.

Variables marked with red were not measured, and thus will not be reported.

Supplementary Table 1: Adherence to the intervention in the $IWT_{support}$, IWT_{only}, and IWT ($IWT_{support}$ + IWT_{only}) groups.

	$IWT_{support}$ group	IWT_{only} group	IWT combined group
InterWalk app data			
Frequency (n/week)			
Duration (min/week)			
Intensity (G)*			
SMS track data			
Total replies, n (%)		n/a	n/a
Total missing replies, n (%)		n/a	n/a
Total answered follow-up questions, n (%)		n/a	n/a
Reported reasons for not using the InterWalk app		n/a	n/a
Illness, n (%)		n/a	n/a
No motivation, n (%)		n/a	n/a
Don't want to walk alone, n (%)		n/a	n/a
Bad weather, n (%)		n/a	n/a
Lack of time, n (%)		n/a	n/a
Due to work, n (%)		n/a	n/a
Other reasons, n (%)		n/a	n/a

Data are mean (SD) or numbers (proportions).

*Intensity is defined as the vector magnitude (G) calculated as the square root of the summed squared accelerations from the x, y and z axes of the on-board accelerometer data sampled (100 Hz) during IWT.

Abbreviations: IWT_{only}, interval walking training, no additional support following the 12-week exercise program; $IWT_{support}$ group, interval walking training, with additional motivational support following the 12-week exercise program; IWT, interval walking training; n/a, not applicable.

Supplementary Table 2: Intention-to-treat analysis of changes from baseline to 12-week intermediate assessment between the IWT group and the StC group*

	IWT group	StC group	Between-group difference	
	LS Mean ± SE	LS Mean ± SE	Difference between means (95% CI)	*P*-value
Primary outcome				
MVPA time (min/day)				
Key secondary outcomes				
SF-12 Physical Component Summary (PCS) (score 0-100)				*N.A.*
SF-12 Mental Component Summary (MCS) (score 0-100)				*N.A.*
VO_{2peak} (ml O_2/min)				*N.A.*
RPAQ self-rated PAEE (kJ/day)				*N.A.*
BREQ-2 RAI (score −24-20)				*N.A.*
Weight (kg)				*N.A.*
Waist circumference (cm)				*N.A.*
Key secondary outcomes with incomplete assessment				
Endurance in the lower extremeties (n/30 sec)	n/a	n/a	n/a	*N.A.*
Body temperature (°C)	n/a	n/a	n/a	*N.A.*
Exploratory secondary outcomes				
Sitting time (min/day)				*N.A.*
LPA time (min/day)				*N.A.*
TPA level (CPM)				*N.A.*
Steps (n/day)				*N.A.*
BMI (kg/m^2)				*N.A.*

Data are LS Means (standard errors) and Difference between means (95% CI's).

*Missing data for the ITT population will be handled using repeated-measures mixed linear models for continuous outcomes.

Abbreviations: IWT, interval walking training; StC, Standard care; LS Means, Least Squares Means; CI, Confidence Interval; MVPA, moderate-to-vigorous physical activity; SF-12, the Short-Form health Survey; N.A., not analyzed; RPAQ, Resent Physical Activity Questionnaire; PAEE, physical activity energy expenditure; BREQ-2, Behavioural regulation in exercise questionnaire-2; RAI, relative autonomy index; VO_{2peak}, peak oxygen consumption; n/a, not applicable; LPA, light physical activity; TPA, total physical activity; CPM, counts per minute; BMI, body mass index.

Variables marked with red were not measured, and thus will not be reported.

Supplementary Table 3: Intention-to-treat analysis of changes from baseline to 52 week follow-up between the IWT_{only} group and the $IWT_{support}$ group*

	StC group	$IWT_{support}$ group	IWT_{only} group	Between-IWT-group difference	
	LS Mean ± SE	LS Mean ± SE	LS Mean ± SE	Difference between means (95% CI)	*P*-value
Primary outcome					
MVPA time (min/day)					
Key secondary outcomes					
SF-12 Physical Component Summary (PCS) (score 0-100)					*N.A.*
SF-12 Mental Component Summary (MCS) (score 0-100)					*N.A.*
VO_{2peak} (ml O_2/min)					*N.A.*
RPAQ self-rated PAEE (kJ/day)					*N.A.*
BREQ-2 RAI (score −24-20)					*N.A.*
Weight (kg)					*N.A.*
Waist circumference (cm)					*N.A.*
Key secondary outcomes with incomplete assessment					
Endurance in the lower extremeties (n/30 sec)	n/a	n/a	n/a	n/a	*N.A.*
Body temperature (°C)	n/a	n/a	n/a	n/a	*N.A.*
Exploratory secondary outcomes					
Sitting time (min/day)					*N.A.*
LPA time (min/day)					*N.A.*
TPA level (CPM)					*N.A.*
Steps (n/day)					*N.A.*
BMI (kg/m^2)					*N.A.*

Data are LS Means (standard errors) and Difference between means (95% CI's).

*Missing data for the ITT population will be handled using repeated-measures mixed linear models for continuous outcomes.

Abbreviations: IWT_{only}, interval walking training, no additional support following the 12-week exercise program; $IWT_{support}$ group, interval walking training, with additional motivational support following the 12-week exercise program; StC, Standard care; LS Means, Least Squares Means; CI, Confidence Interval; MVPA, moderate-to-vigorous physical activity; SF-12, the Short-Form health Survey; N.A., not analyzed; RPAQ, Resent Physical Activity Questionnaire; PAEE, physical activity energy expenditure; BREQ-2, Behavioural regulation in exercise questionnaire-2; RAI, relative autonomy index; VO_{2peak}, peak oxygen consumption; n/a, not applicable; LPA, light physical activity; TPA, total physical activity; CPM, counts per minute; BMI, body mass index.

Variables marked with red were not measured, and thus will not be reported.

Supplementary Table 4: Sensitivity analyses of changes in MVPA time from baseline to 52-week follow-up between the IWT group and the StC group: (1) Per-protocol analysis; (2) Intention-to-treat analysis with non-responder (BOCF) imputation; (3) Intention-to-treat analysis with the subsample of participants whose intervention period did not overlap time of technological malfunction of the InterWalk application; (4) Intention-to-treat analysis with alternative cut-off at 3000 CPM (i.e. corresponding to forced walking); and (5) Intention-to-treat analysis with daytime criteria for accelerometer data (i.e. ≥3 days of ≥14 h of daytime measurement (6 am to 22 pm).

	IWT group	StC group	Between-group difference
	LS Mean ± SE	LS Mean ± SE	Difference between means (95% CI)
(1) Per-protocol			
MVPA time (min/day)			
(2) Non-responder, single-step imputation			
MVPA time (min/day)			
(3) Subsample with app			
MVPA time (min/day)			
(4) Cut-off, 3000 CPM			
MVPA time (min/day)			
(5) Daytime criteria			
MVPA time (min/day)			

Data are LS Means (standard errors) and Difference between means (95% CI's).

Abbreviations: IWT, interval walking training; StC, Standard care; LS Means, Least Squares Means; CI, Confidence Interval; MVPA, moderate-to-vigorous physical activity; BOCF, baseline observation carried forward; CPM, counts per minute.

REFERENCES

1. American Diabetes Association, *5. Facilitating Behavior Change and Well-being to Improve Health Outcomes: Standards of Medical Care in Diabetes-2020.* Diabetes Care, 2020. **43**(Suppl 1): p. S48-S65.
2. Umpierre, D., et al., *Physical activity advice only or structured exercise training and association with HbA1c levels in type 2 diabetes: a systematic review and meta-analysis.* JAMA, 2011. **305**(17): p. 1790-9.
3. Saeedi, P., et al., *Global and regional diabetes prevalence estimates for 2019 and projections for 2030 and 2045: Results from the International Diabetes Federation Diabetes Atlas, 9(th) edition.* Diabetes Res Clin Pract, 2019. **157**: p. 107843.
4. Nemoto, K., et al., *Effects of high-intensity interval walking training on physical fitness and blood pressure in middle-aged and older people.* Mayo Clin Proc, 2007. **82**(7): p. 803-11.
5. Masuki, S., M. Morikawa, and H. Nose, *Interval Walking Training Can Increase Physical Fitness in Middle-Aged and Older People.* Exerc Sport Sci Rev, 2017. **45**(3): p. 154-162.
6. Karstoft, K., et al., *The effects of free-living interval-walking training on glycemic control, body composition, and physical fitness in type 2 diabetic patients: a randomized, controlled trial.* Diabetes Care, 2013. **36**(2): p. 228-36.
7. Ried-Larsen, M., et al., *Implementation of interval walking training in patients with type 2 diabetes in Denmark: rationale, design, and baseline characteristics.* Clin Epidemiol, 2016. **8**: p. 201-9.
8. Valentiner, L.S., et al., *Long-term effect of smartphone-delivered Interval Walking Training on physical activity in patients with type 2 diabetes: protocol for a parallel group single-blinded randomised controlled trial.* BMJ Open, 2017. **7**(4): p. e014036.
9. Brinklov, C.F., et al., *Criterion validity and reliability of a smartphone delivered sub-maximal fitness test for people with type 2 diabetes.* BMC Sports Sci Med Rehabil, 2016. **8**: p. 31.
10. Cao, J. and S. Zhang, *Multiple comparison procedures.* JAMA, 2014. **312**(5): p. 543-4.
11. Hollis, S. and F. Campbell, *What is meant by intention to treat analysis? Survey of published randomised controlled trials.* BMJ, 1999. **319**(7211): p. 670-4.
12. Detry, M.A. and Y. Ma, *Analyzing Repeated Measurements Using Mixed Models.* JAMA, 2016. **315**(4): p. 407-8.
13. Freedson, P.S., E. Melanson, and J. Sirard, *Calibration of the Computer Science and Applications, Inc. accelerometer.* Med Sci Sports Exerc, 1998. **30**(5): p. 777-81.
14. Ware, J., Jr., M. Kosinski, and S.D. Keller, *A 12-Item Short-Form Health Survey: construction of scales and preliminary tests of reliability and validity.* Med Care, 1996. **34**(3): p. 220-33.
15. Diaz-Arribas, M.J., et al., *Minimal Clinically Important Difference in Quality of Life for Patients With Low Back Pain.* Spine (Phila Pa 1976), 2017. **42**(24): p. 1908-1916.
