# Supplementary material for: The Effects of a Lifestyle Intervention Supported by the InterWalk Smartphone App on Increasing Physical Activity Among Persons With Type 2 Diabetes: Parallel-Group, Randomized Trial
Source: JMIR Mhealth Uhealth. 2022 Sep 28;10(9):e30602. doi: 10.2196/30602 (PMC9557767; doi:10.2196/30602)
Supplement: Multimedia Appendix 2 [file mhealth_v10i9e30602_app2.docx]

|  | StC group | IWT combined group | Total | IWT_only_ group | IWT_support_ group |
| --- | --- | --- | --- | --- | --- |
| ***Baseline*** |  |  |  |  |  |
| No. of days (n)^a^ | 7 (7; 7) | 7 (7; 7) | 7 (7; 7) | 7 (7; 8) | 7 (7; 7) |
| Wear time |  |  |  |  |  |
| *Back (h/day)*^a^ | 23.3 (23.3; 23.3) | 23.3 (23.3; 23.3) | 23.3 (23.3; 23.3) | 23.3 (23.2; 23.3) | 23.3 (23.3; 23.3) |
| *Thigh (h/day)*^b^ | 23.3 (23.0; 23.3) | 23.3 (23.1; 23.3) | 23.3 (23.1; 23.3) | 23.3 (22.8; 23.3) | 23.3 (23.2; 23.3) |
| Nonwear time |  |  |  |  |  |
| *Back (min/day)*^a^ | 0.0 (0.0; 0.0) | 0.0 (0.0; 0.0) | 0.0 (0.0; 0.0) | 0.0 (0.0; 0.0) | 0.0 (0.0; 0.0) |
| *Thigh (min/day)*^b^ | 0.0 (0.0; 1.6) | 0.0 (0.0; 4.9) | 0.0 (0.0; 4.3) | 0.0 (0.0; 12.0) | 0.0 (0.0; 3.6) |
| ***12-weeks follow-up*** |  |  |  |  |  |
| No. of days (n)^c^ | 7 (7; 8) | 7 (7; 7) | 7 (7; 7) | 7 (6; 7) | 7 (7; 7) |
| Wear time |  |  |  |  |  |
| *Back (h/day)*^c^ | 23.3 (23.2; 23.4) | 23.3 (23.3; 23.3) | 23.3 (23.3; 23.4) | 23.3 (23.2; 23.3) | 23.3 (23.3; 23.3) |
| *Thigh (h/day)*^d^ | 23.3 (23.2; 23.3) | 23.3 (23.1; 23.3) | 23.3 (23.1; 23.3) | 23.3 (23.2; 23.3) | 23.3 (22.3; 23.3) |
| Nonwear time |  |  |  |  |  |
| *Back (min/day)*^c^ | 0.0 (0.0; 3.6) | 0.0 (0.0; 0.0) | 0.0 (0.0; 0.0) | 0.0 (0.0; 0.0) | 0.0 (0.0; 0.0) |
| *Thigh (min/day)*^d^ | 0.0 (0.0; 7.4) | 0.0 (0.0; 3.1) | 0.0 (0.0; 7.1) | 0.0 (0.0; 0.0) | 0.0 (0.0; 52.3) |
| ***52-weeks follow-up*** |  |  |  |  |  |
| No. of days (n)^e^ | 7 (7; 8) | 7 (7; 8) | 7 (7; 8) | 7 (7; 8) | 7 (7; 8) |
| Wear time |  |  |  |  |  |
| *Back (h/day)*^e^ | 23.3 (23.3; 23.4) | 23.3 (23.2; 23.4) | 23.3 (23.3; 23.4) | 23.3 (23.2; 23.4) | 23.3 (23.3; 23.4) |
| *Thigh (h/day)*^f^ | 23.3 (23.0; 23.3) | 23.3 (23.2; 23.3) | 23.3 (23.1; 23.3) | 23.3 (23.2; 23.3) | 23.3 (23.1; 23.3) |
| Nonwear time |  |  |  |  |  |
| *Back (min/day)*^e^ | 0.0 (0.0; 0.0) | 0.0 (0.0; 5.7) | 0.0 (0.0; 1.7) | 0.0 (0.0; 1.7) | 0.0 (0.0; 6.3) |
| *Thigh (min/day)*^f^ | 0.0 (0.0; 8.7) | 0.0 (0.0; 4.9) | 0.0 (0.0; 7.5) | 0.0 (0.0; 1.3) | 0.0 (0.0; 10.3) |

Data are medians (25^th^; 75^th^ percentile). On day 1 accelerometer measures began at 5 a.m. Criteria for inclusion was ≥3 accepted days of <2h non-wear.

^a^StC, n=70; IWT combined, n=130; Total, n=200; IWT_only_, n=66; IWT_support_, n=64

^b^StC, n=68; IWT combined, n=127; Total, n=195; IWT_only_, n=66; IWT_support_, n=61

^c^StC, n=55; IWT combined, n=94; Total, n=149; IWT_only_, n=44; IWT_support_, n=50

^d^StC, n=53; IWT combined, n=94; Total, n=147; IWT_only_, n=44; IWT_support_, n=50

^e^StC, n=42; IWT combined, n=80; Total, n=122; IWT_only_, n=36; IWT_support_, n=44

^f^StC, n=42; IWT combined, n=78; Total, n=120; IWT_only_, n=36; IWT_support_, n=42.

Abbreviations: IWT, interval walking training; StC, Standard care; IWT_support_ group, interval walking training, with additional motivational support following the 12-week exercise program; IWT_only_, interval walking training, no additional support following the 12-week exercise program.
