# Supplementary material for: The Effects of a Lifestyle Intervention Supported by the InterWalk Smartphone App on Increasing Physical Activity Among Persons With Type 2 Diabetes: Parallel-Group, Randomized Trial
Source: JMIR Mhealth Uhealth. 2022 Sep 28;10(9):e30602. doi: 10.2196/30602 (PMC9557767; doi:10.2196/30602)
Supplement: Multimedia Appendix 4 [file mhealth_v10i9e30602_app4.docx]

|  | IWT combined group | IWT_only_ group | IWT_support_ group |
| --- | --- | --- | --- |
| ***InterWalk app data*** |  |  |  |
| 0-52 weeks |  |  |  |
| *Frequency (n/week)*^a^ | 0.3 (0.1; 0.7) | 0.3 (0.1; 0.6) | 0.3 (0.1; 0.7) |
| *Duration (min/week)*^a^ | 11.4 (4.1; 22.3) | 13.0 (4.2; 21.2) | 9.7 (3.8; 23.4) |
| *Intensity (G)*^a,d^ | 0.17 (0.11; 0.23) | 0.16 (0.10; 0.21) | 0.18 (0.13; 0.23) |
| 0-12 weeks |  |  |  |
| *Frequency (n/week)*^b^ | 1.1 (0.4; 1.9) | 1.1 (0.4; 2.0) | 1.1 (0.5; 1.8) |
| *Duration (min/week)*^b^ | 38.0 (16.2; 69.9) | 40.8 (17.1; 69.9) | 37.6 (14.7; 68.9) |
| *Intensity (G)*^b,d^ | 0.16 (0.12; 0.22) | 0.16 (0.11; 0.22) | 0.18 (0.13; 0.23) |
| 13-52 weeks |  |  |  |
| *Frequency (n/week)*^c^ | 0.2 (0.1; 0.6) | 0.2 (0.1; 0.7) | 0.3 (0.1; 0.6) |
| *Duration (min/week)*^c^ | 7.7 (2.2; 29.0) | 6.5 (2.2; 30.4) | 8.8 (2.3; 24.9) |
| *Intensity (G)*^c,d^ | 0.17 (0.10; 0.22) | 0.16 (0.10; 0.21) | 0.17 (0.12; 0.23) |
| ***SMS survey data*** |  |  |  |
| Total replies, n (%) | n/a | n/a | 1536 (78.8) |
| Total missing replies, n (%) | n/a | n/a | 414 (21.2) |
| Total answered follow-up questions, n (%) | n/a | n/a | 418 (97.0) |
| Weekly use of the InterWalk app |  |  |  |
| *I have not used the app this week, n (%)* | n/a | n/a | 432 (28.1) |
| *1-2 times/week, n (%)* | n/a | n/a | 510 (33.2) |
| *3 times/week, n (%)* | n/a | n/a | 332 (21.6) |
| *More than 3 times/week, n (%)* | n/a | n/a | 262 (17.1) |
| Reported reasons for not using the InterWalk app |  |  |  |
| *Illness, n (%)* | n/a | n/a | 158 (36.7) |
| *No motivation, n (%)* | n/a | n/a | 14 (3.2) |
| *Don’t want to walk alone, n (%)* | n/a | n/a | 2 (0.5) |
| *Bad weather, n (%)* | n/a | n/a | 13 (3.0) |
| *Lack of time, n (%)* | n/a | n/a | 37 (8.6) |
| *Due to work, n (%)* | n/a | n/a | 44 (10.2) |
| *Other reasons, n (%)* | n/a | n/a | 150 (34.8) |

Data are medians (25^th^; 75^th^ percentile) or numbers (proportions).

^a^IWT combined, n=110; IWT_only_, n=52; IWT_support_, n=58

^b^IWT combined, n=108; IWT_only_, n=52; IWT_support_, n=56

^c^IWT combined, n=56; IWT_only_, n=23; IWT_support_, n=33

^d^Intensity is defined as the vector magnitude (G) calculated as the square root of the summed squared accelerations from the x, y and z axes of the on-board accelerometer data sampled (100 Hz) during IWT.

Abbreviations: IWT_support_ group, interval walking training, with additional motivational support following the 12-week exercise program; IWT_only_, interval walking training, no additional support following the 12-week exercise program; IWT, interval walking training; n/a, not applicable.
