# Supplementary material for: The Effects of a Lifestyle Intervention Supported by the InterWalk Smartphone App on Increasing Physical Activity Among Persons With Type 2 Diabetes: Parallel-Group, Randomized Trial
Source: JMIR Mhealth Uhealth. 2022 Sep 28;10(9):e30602. doi: 10.2196/30602 (PMC9557767; doi:10.2196/30602)
Supplement: Multimedia Appendix 5 [file mhealth_v10i9e30602_app5.docx]

|  | | | | | |  |
| --- | --- | --- | --- | --- | --- | --- |
|  | Week 16 | Week 20 | Week 28 | Week 40 | Total | |
| Number (%) interviews complete | 40 (85) | 36 (77) | 37 (79) | 35 (75) | 148 (79) | |
|  |  |  |  |  |  | |
| Number (%) of participants with completed interviews (4/3/2/1/0) |  |  |  |  | 27 (58)/11 (23)/2 (4)/3 (6)/4 (9) | |
|  |  |  |  |  |  | |
| Number (%) of participants reporting performing IWT with other participants* | 12 (26) | 9(19) | 12(26) | 10 (21) |  | |
|  |  |  |  |  |  | |
| Number (%) of participants reporting performing IWT with the Danish Diabetes Association (N (%)) | 6 (13) | 5 (11) | 5 (11) | 5 (11) |  | |
|  |  |  |  |  |  | |
| Number (%) of participants with goals explicitly including IWT | 20 (43) | 19 (40) | 17 (36) | 15 (32) |  | |
|  |  |  |  |  |  | |
| Number (%) of participants expressing technical issues with the InterWalk app (N (%))# | 2 (4) | 4 (9) | 1 (2) | 3 (6) |  | |
|  |  |  |  |  |  | |
| IWT Interval walking traning  Denominator = 47  *including with the diabetes association  # might be higher as due to the substantial loss to follow-up. The drop might have been caused by technical issues | | | | | |  |
