# Supplementary material for: The Effects of a Lifestyle Intervention Supported by the InterWalk Smartphone App on Increasing Physical Activity Among Persons With Type 2 Diabetes: Parallel-Group, Randomized Trial
Source: JMIR Mhealth Uhealth. 2022 Sep 28;10(9):e30602. doi: 10.2196/30602 (PMC9557767; doi:10.2196/30602)
Supplement: Multimedia Appendix 6 [file mhealth_v10i9e30602_app6.docx]

|  | StC group | IWT group | Between-group difference | |
| --- | --- | --- | --- | --- |
|  | LS Mean (95% CI) | LS Mean (95% CI) | Difference between means (95% CI) | *P* value |
| ***Primary outcome*** |  |  |  |  |
| MVPA time (min/day)^a^ | 1.3 (-3.5 to 6.2) | 3.6 (0.2 to 6.9) | 2.2 (-4.6 to 9.0) | .52 |
| ***Key secondary outcomes*** |  |  |  |  |
| SF-12 Physical Component Summary (PCS) (score 0-100) | 0.6 (-1.2 to 2.3) | 4.2 (3.0 to 5.4) | 3.7 (1.2 to 6.1) | *N.A.* |
| SF-12 Mental Component Summary (MCS) (score 0-100) | 4.0 (1.8 to 6.3) | 2.0 (0.4 to 3.5) | -2.1 (-5.2 to 1.1) | *N.A.* |
| VO_2peak_ (ml O_2_/min)^b^ | 96.5 (-94.8 to 287.8) | 98.1 (17.1 to 179.2) | 1.6 (-227.7 to 230.9) | *N.A.* |
| RPAQ self-rated PAEE (kJ/ kg/day)^c^ | 12.8 (-9.6 to 35.2) | -0.9 (-16.0 to 14.1) | -13.7 (-45.0 to 17.6) | *N.A.* |
| BREQ-2 RAI (score −24-20) | 2.3 (0.8 to 3.7) | 2.3 (1.3 to 3.2) | 0.0 (-2.0 to 2.0) | *N.A.* |
| Weight (kg)^d^ | -2.2 (-3.3 to -1.0) | -2.2 (-3.0 to -1.5) | -0.1 (-1.7 to 1.5) | *N.A.* |
| Waist circumference (cm)^e^ | -3.2 (-4.5 to -1.9) | -2.9 (-3.7 to -2.0) | 0.3 (-1.5 to 2.1) | *N.A.* |
| ***Exploratory secondary outcomes*** |  |  |  |  |
| Sitting time (min/day)^f^ | -8.1 (-47.1 to 31.0) | -9.0 (-35.7 to 17.6) | -1.0 (-54.9 to 53.0) | *N.A.* |
| LPA time (min/day)^a^ | -12.3 (-21.3 to -3.4) | 7.8 (1.6 to 14.0) | 20.1 (7.5 to 32.7) | *N.A.* |
| TPA level (CPM)^a^ | -7.8 (-34.4 to 18.9) | 22.2 (3.9 to 40.5) | 30.0 (-7.7 to 67.7) | *N.A.* |
| Steps (n/day)^a^ | -296 (-910 to 317) | 827 (407 to 1248) | 1124 (255 to 1992) | *N.A.* |
| BMI (kg/m^2^)^d^ | -0.7 (-1.1 to -0.4) | -0.8 (-1.0 to -0.5) | 0.0 (-0.6 to 0.5) | *N.A.* |

Data are LS Means (95% CI’s) and Difference between means (95% CI’s).

^a^n=200; ^b^n=56; ^c^n=209; ^d^n=212; ^e^n=211; ^f^n=195

Abbreviations: StC, Standard care; IWT, interval walking training; LS Mean, Least Squares Mean; CI, Confidence Interval; MVPA, moderate-to-vigorous physical activity; SF-12, the Short-Form Health Survey; N.A., not analyzed; VO_2peak_, peak oxygen consumption; RPAQ, Recent Physical Activity Questionnaire; PAEE, physical activity energy expenditure; BREQ-2, Behavioral Regulation in Exercise Questionnaire-2; RAI, Relative Autonomy Index; LPA, light physical activity; TPA, total physical activity; CPM, counts per minute; BMI, body mass index.
