# Supplementary material for: The Effects of a Lifestyle Intervention Supported by the InterWalk Smartphone App on Increasing Physical Activity Among Persons With Type 2 Diabetes: Parallel-Group, Randomized Trial
Source: JMIR Mhealth Uhealth. 2022 Sep 28;10(9):e30602. doi: 10.2196/30602 (PMC9557767; doi:10.2196/30602)
Supplement: Multimedia Appendix 7 [file mhealth_v10i9e30602_app7.docx]

|  | StC group | IWT group | Between-group difference |
| --- | --- | --- | --- |
|  | LS Mean (95% CI) | LS Mean (95% CI) | Difference between means (95% CI) |
| ***(1) Per-protocol*** |  |  |  |
| MVPA time (min/day) | n/a | n/a | n/a |
| ***(2) Non-responder, single-step imputation*** | n=70 | n=130 |  |
| MVPA time (min/day) | 0.5 (-3.3 to 4.3) | -0.2 (-2.7 to 2.3) | -0.7 (-6.0 to 4.6) |
| ***(3) Subsample with app*** | n=70 | n=56 |  |
| MVPA time (min/day) | 2.7 (-2.1 to 7.5) | -2.2 (-8.1 to 3.8) | -4.9 (-13.8 to 4.1) |
| ***(4) Daytime criteria*** | n=70 | n=130 |  |
| MVPA time (min/day) | 0.9 (-4.4 to 6.2) | 0.0 (-3.6 to 3.7) | -0.9 (-8.3 to 6.5) |
| ***(5) Cut-off, 3000 CPM*** | n=70 | n=130 |  |
| Forced walking time (min/day) | 1.0 (-3.2 to 5.2) | 0.1 (-2.7 to 3.0) | -0.9 (-6.7 to 5.0) |

Data are LS Means (95% CI’s) and Difference between means (95% CI’s).

Abbreviations: StC, Standard care; IWT, interval walking training; LS Mean, Least Squares Mean; CI, Confidence Interval; MVPA, moderate-to-vigorous physical activity; n/a, not applicable; CPM, counts per minute.
