# Supplementary material for: The Effects of a Lifestyle Intervention Supported by the InterWalk Smartphone App on Increasing Physical Activity Among Persons With Type 2 Diabetes: Parallel-Group, Randomized Trial
Source: JMIR Mhealth Uhealth. 2022 Sep 28;10(9):e30602. doi: 10.2196/30602 (PMC9557767; doi:10.2196/30602)
Supplement: Multimedia Appendix 8 [file mhealth_v10i9e30602_app8.docx]

**Figure S2:** *Post hoc* linear regression analysis of the dose response association over 52 weeks between (a) change in MVPA (min/day) and change in SF-12 PCS score; (b) IWT duration (min/week) and change in SF-12 PCS score; (c) change in MVPA (min/day) and change in waist circumference (cm); and (d) IWT duration (min/week) and change in waist circumference (cm).

*r* = 0.04, *P* = .70

*r* = 0.07, *P* = .57

*r* = -0.21, *P* = .03

*r* = -0.30, *P* = .01
