# Supplementary material for: The Effects of a Lifestyle Intervention Supported by the InterWalk Smartphone App on Increasing Physical Activity Among Persons With Type 2 Diabetes: Parallel-Group, Randomized Trial
Source: JMIR Mhealth Uhealth. 2022 Sep 28;10(9):e30602. doi: 10.2196/30602 (PMC9557767; doi:10.2196/30602)
Supplement: Multimedia Appendix 9 [file mhealth_v10i9e30602_app9.docx]

|  | StC group | IWT_only_ group | IWT_support_ group | Between-IWT-group difference | |
| --- | --- | --- | --- | --- | --- |
|  | LS Mean (95% CI) | LS Mean (95% CI) | LS Mean (95% CI) | Difference between means (95% CI) | *P* value |
| ***Primary outcome*** |  |  |  |  |  |
| MVPA time (min/day)^a^ | -0.9 (-4.9 to 3.2) | -1.5 (-5.9 to 2.9) | 2.6 (-1.5 to 6.7) | 4.1 (-1.9 to 10.1) | .18 |
| ***Key secondary outcomes*** |  |  |  |  |  |
| SF-12 Physical Component Summary (PCS) (score 0-100) | 0.2 (-1.3 to 1.6) | 4.4 (2.9 to 6.0) | 4.0 (2.6 to 5.4) | -0.4 (-2.5 to 1.7) | *N.A.* |
| SF-12 Mental Component Summary (MCS) (score 0-100) | 1.4 (-0.5 to 3.3) | 1.5 (-0.6 to 3.6) | 2.4 (0.5 to 4.3) | 0.9 (-2.0 to 3.7) | *N.A.* |
| VO_2peak_ (ml O_2_/min)^b^ | -7.5 (-98.0 to 82.9) | 55.8 (-36.8 to 148.3) | 97.8 (23.1 to 172.5) | 42.0 (-76.9 to 160.9) | *N.A.* |
| RPAQ self-rated PAEE (kJ/ kg/day)^c^ | 9.9 (-8.6 to 28.4) | -1.5 (-21.7 to 18.6) | -15.9 (-34.4 to 2.6) | -14.4 (-41.7 to 12.9) | *N.A.* |
| BREQ-2 RAI (score −24-20) | 1.7 (0.5 to 2.8) | 2.7 (1.5 to 4.0) | 2.1 (0.9 to 3.2) | -0.7 (-2.4 to 1.1) | *N.A.* |
| Weight (kg)^d^ | -0.2 (-1.1 to 0.7) | -1.7 (-2.6 to -0.7) | -1.4 (-2.3 to -0.5) | 0.3 (-1.1 to 1.6) | *N.A.* |
| Waist circumference (cm)^e^ | -0.8 (-1.9 to 0.2) | -3.0 (-4.1 to -1.9) | -3.3 (-4.3 to -2.3) | -0.3 (-1.8 to 1.2) | *N.A.* |
| ***Exploratory secondary outcomes*** |  |  |  |  |  |
| Sitting time (min/day)^f^ | 39.2 (5.3 to 73.1) | -5.3 (-41.5 to 30.9) | -20.0 (-55.3 to 15.3) | -14.7 (-65.3 to 35.9) | *N.A.* |
| LPA time (min/day)^a^ | -9.8 (-17.4 to -2.2) | -1.4 (-9.6 to 6.8) | -10.9 (-18.6 to -3.2) | -9.5 (-20.8 to 1.7) | *N.A.* |
| TPA level (CPM)^a^ | -13.1 (-35.3 to 9.2) | -0.7 (-24.7 to 23.4) | 8.1 (-14.5 to 30.7) | 8.8 (-24.2 to 41.8) | *N.A.* |
| Steps (n/day)^a^ | -420 (-929 to 89) | 74 (-476 to 624) | 230 (-286 to 746) | 156 (-599 to 910) | *N.A.* |
| BMI (kg/m^2^)^d^ | -0.1 (-0.4 to 0.2) | -0.6 (-0.9 to -0.2) | -0.5 (-0.8 to -0.2) | 0.1 (-0.3 to 0.6) | *N.A.* |

Data are LS Means (95% CI’s) and Difference between means (95% CI’s).

^a^n=200; ^b^n=54; ^c^n=209; ^d^n=212; ^e^n=211; ^f^n=195

Abbreviations: StC, Standard care; IWT_only_, interval walking training, no additional support following the 12-week exercise program; IWT_support_ group, interval walking training, with additional motivational support following the 12-week exercise program; LS Mean, Least Squares Mean; CI, Confidence Interval; MVPA, moderate-to-vigorous physical activity; SF-12, the Short-Form Health Survey; N.A., not analyzed; VO_2peak_, peak oxygen consumption; RPAQ, Recent Physical Activity Questionnaire; PAEE, physical activity energy expenditure; BREQ-2, Behavioral Regulation in Exercise Questionnaire-2; RAI, Relative Autonomy Index; LPA, light physical activity; TPA, total physical activity; CPM, counts per minute; BMI, body mass index.
