# Supplementary material for: The Effects of a Lifestyle Intervention Supported by the InterWalk Smartphone App on Increasing Physical Activity Among Persons With Type 2 Diabetes: Parallel-Group, Randomized Trial
Source: JMIR Mhealth Uhealth. 2022 Sep 28;10(9):e30602. doi: 10.2196/30602 (PMC9557767; doi:10.2196/30602)
Supplement: Multimedia Appendix 11 [file mhealth_v10i9e30602_app11.docx]

**Table S7:** Baseline demographic and clinical characteristics among attenders and nonattenders at 52-week follow-up

|  | Attenders (n=130) | Nonattenders (n=84) | Total |
| --- | --- | --- | --- |
| Group allocation |  |  |  |
| *StC group, n (%)* | 45 (34.6) | 29 (34.5) | 74 (34.6) |
| *IWT_only_ group, n (%)* | 38 (29.2) | 31 (36.9) | 69 (32.2) |
| *IWT_support_ group, n (%)* | 47 (36.2) | 24 (28.6) | 71 (33.2) |
| ***Demographic and clinical characteristics*** |  |  |  |
| Sex: |  |  |  |
| *Male, n (%)* | 74 (56.9) | 54 (64.3) | 128 (59.8) |
| *Female, n (%)* | 56 (43.1) | 30 (35.7) | 86 (40.2) |
| Age (years) | 59.7 (11.1) | 59.5 (9.7) | 59.6 (10.6) |
| T2D duration^a^: |  |  |  |
| *T2D duration (years)*^†^ | 3.8 (0.2; 10.0) | 1.7 (0.1; 7.0) | 2.6 (0.2; 8.6) |
| *≤5 years, n (%)* | 65 (54.6) | 54 (69.2) | 119 (60.4) |
| *>5 years, n (%)* | 54 (45.4) | 24 (30.8) | 78 (39.6) |
| Alcohol consumption^b^: |  |  |  |
| *Within the recommended levels, n (%)* | 126 (97.7) | 72 (85.7) | 198 (93.0) |
| *Above recommendations, n (%)* | 3 (2.3) | 12 (14.3) | 15 (7.0) |
| Smoking habits: |  |  |  |
| *Smoker, n (%)* | 22 (16.9) | 27 (32.1) | 49 (22.9) |
| *Nonsmoker, n (%)* | 108 (83.1) | 57 (67.9) | 165 (77.1) |
| Highest level of education^c^: |  |  |  |
| *ISCED-2011 levels 0-4, n (%)* | 65 (51.6) | 49 (59.0) | 114 (54.6) |
| *ISCED-2011 levels 5-8, n (%)* | 61 (48.4) | 34 (41.0) | 95 (45.4) |
| Civil status: |  |  |  |
| *Single, divorced or widowed, n (%)* | 61 (46.9) | 39 (46.4) | 100 (46.7) |
| *Married or cohabiting, n (%)* | 69 (53.1) | 45 (53.6) | 114 (53.3) |
| Height (cm)^d^ | 171.7 (8.9) | 173.1 (9.1) | 172.2 (9.0) |
| ***Physical activity and ftiness*** |  |  |  |
| MVPA time (min/day)^†,e^ | 33.3 (19.8; 48.4) | 26.8 (12.9; 47.0) | 30.2 (16.9; 48.1) |
| Sitting time (min/day)^h^ | 534.0 (150.5) | 542.9 (177.8) | 537.4 (161.0) |
| LPA time (min/day)^e^ | 138.7 (49.4) | 132.0 (50.7) | 136.1 (49.9) |
| TPA level (CPM)^†,e^ | 227.5 (159.1; 310.3) | 182.8 (120.3; 319.2) | 211.5 (140.9; 313.8) |
| Steps (n/day)^†,e^ | 4465 (3078; 6188) | 3816 (2339; 5548) | 4100 (2794; 5986) |
| VO_2peak_ (ml O_2_/min)^f^ | 1781 (456) | 1805 (519) | 1791 (481) |
| ***Self-reported measures*** |  |  |  |
| SF-12 Physical Component Summary (PCS) (score 0-100) | 42.0 (9.3) | 39.8 (11.4) | 41.1 (10.2) |
| SF-12 Mental Component Summary (MCS) (score 0-100)^†^ | 51.3 (43.3; 57.3) | 46.1 (38.1; 55.7) | 50.1 (40.1; 56.9) |
| RPAQ self-rated PAEE (kJ/ kg/day)^†,c^ | 143.3 (100.6; 217.5) | 145.9 (82.3; 235.7) | 143.8 (92.9; 226.4) |
| BREQ-2 RAI (score −24-20)^†^ | 7.6 (3.3; 11.7) | 7.0 (4.0; 10.6) | 7.4 (3.5; 11.3) |
| ***Exploratory secondary outcomes*** |  |  |  |
| Weight (kg)^†,d^ | 98 (85; 110) | 101 (89; 120) | 99 (87; 115) |
| Waist circumference (cm)^g^ | 114.9 (12.7) | 118.3 (15.9) | 116.2 (14.1) |
| BMI (kg/m^2^)^d^ | 33.5 (5.2) | 34.7 (6.5) | 34.0 (5.7) |

Data are means (standard deviations), ^†^medians (interquartile ranges), or numbers (proportions).

^a^n=197; ^b^n=213; ^c^n=209; ^d^n=212; ^e^n=200; ^f^n=124; ^g^n=211; ^h^n=195

Abbreviations: StC, Standard care; IWT, interval walking training; T2D, type 2 diabetes; ISCED-2011, International Standard Classification of Education 2011; MVPA, moderate-to-vigorous physical activity; SF-12, the Short-Form Health Survey; VO_2peak_, peak oxygen consumption; RPAQ, Recent Physical Activity Questionnaire; PAEE, physical activity energy expenditure; BREQ-2, Behavioral Regulation in Exercise Questionnaire-2; RAI, Relative Autonomy Index; LPA, light physical activity; TPA, total physical activity; CPM, counts per minute; BMI, body mass index.
