# Supplementary material for: The Effects of a Lifestyle Intervention Supported by the InterWalk Smartphone App on Increasing Physical Activity Among Persons With Type 2 Diabetes: Parallel-Group, Randomized Trial
Source: JMIR Mhealth Uhealth. 2022 Sep 28;10(9):e30602. doi: 10.2196/30602 (PMC9557767; doi:10.2196/30602)
Supplement: Multimedia Appendix 12 [file mhealth_v10i9e30602_app12.pdf]

# CONSORT-EHEALTH (V 1.6.1) - Submission/Publication Form

The CONSORT-EHEALTH checklist is intended for authors of randomized trials evaluating web-based and Internet-based applications/interventions, including mobile interventions, electronic games (incl multiplayer games), social media, certain telehealth applications, and other interactive and/or networked electronic applications. Some of the items (e.g. all subitems under item 5 - description of the intervention) may also be applicable for other study designs.

The goal of the CONSORT EHEALTH checklist and guideline is to be
a) a guide for reporting for authors of RCTs,
b) to form a basis for appraisal of an ehealth trial (in terms of validity)

CONSORT-EHEALTH items/subitems are MANDATORY reporting items for studies published in the Journal of Medical Internet Research and other journals / scientific societies endorsing the checklist.

Items numbered 1., 2., 3., 4a., 4b etc are original CONSORT or CONSORT-NPT (non-pharmacologic treatment) items.
Items with Roman numerals (i., ii, iii, iv etc.) are CONSORT-EHEALTH extensions/clarifications.

As the CONSORT-EHEALTH checklist is still considered in a formative stage, we would ask that you also RATE ON A SCALE OF 1-5 how important/useful you feel each item is FOR THE PURPOSE OF THE CHECKLIST and reporting guideline (optional).

Mandatory reporting items are marked with a red *.
In the textboxes, either copy & paste the relevant sections from your manuscript into this form - please include any quotes from your manuscript in QUOTATION MARKS,
or answer directly by providing additional information not in the manuscript, or elaborating on why the item was not relevant for this study.

YOUR ANSWERS WILL BE PUBLISHED AS A SUPPLEMENTARY FILE TO YOUR PUBLICATION IN JMIR AND ARE CONSIDERED PART OF YOUR PUBLICATION (IF ACCEPTED).
Please fill in these questions diligently. Information will not be copyedited, so please use proper spelling and grammar, use correct capitalization, and avoid abbreviations.

DO NOT FORGET TO SAVE AS PDF _AND_ CLICK THE SUBMIT BUTTON SO YOUR ANSWERS ARE IN OUR DATABASE !!!

Citation Suggestion (if you append the pdf as Appendix we suggest to cite this paper in the caption):
Eysenbach G, CONSORT-EHEALTH Group
CONSORT-EHEALTH: Improving and Standardizing Evaluation Reports of Web-based and Mobile Health Interventions
J Med Internet Res 2011;13(4):e126

URL: http://www.jmir.org/2011/4/e126/
doi: 10.2196/jmir.1923
PMID: 22209829

idakaer@gmail.com (deles ikke) Skift konto

Kladden blev gemt

*Skal udfyldes

Your name *

First Last

Ida Thorsen

Primary Affiliation (short), City, Country *

University of Toronto, Toronto, Canada

Centre for Physical Activity Research, Copenha

Your e-mail address *

abc@gmail.com

ida.kaer.thorsen@regionh.dk

Title of your manuscript *

Provide the (draft) title of your manuscript.

The Effects of a Lifestyle Intervention Supported by the InterWalk Smartphone App on Increasing Physical Activity Among Persons With Type 2 Diabetes: Parallel-Group, Randomized Trial

Name of your App/Software/Intervention *

If there is a short and a long/alternate name, write the short name first and add the long name in brackets.

InterWalk

Evaluated Version (if any)

e.g. "V1", "Release 2017-03-01", "Version 2.0.27913"

Versions 8 and 9

Language(s) *

What language is the intervention/app in? If multiple languages are available, separate by comma (e.g. "English, French")

Danish

URL of your Intervention Website or App

e.g. a direct link to the mobile app on app in appstore (itunes, Google Play), or URL of the website. If the intervention is a DVD or hardware, you can also link to an Amazon page.

www.aktivsundhed.dk

URL of an image/screenshot (optional)

Dit svar

Accessibility *

Can an enduser access the intervention presently?

- ○ access is free and open
- ○ access only for special usergroups, not open
- ○ access is open to everyone, but requires payment/subscription/in-app purchases
- ◉ app/intervention no longer accessible
- ○ Andet:

Primary Medical Indication/Disease/Condition *

e.g. "Stress", "Diabetes", or define the target group in brackets after the condition, e.g. "Autism (Parents of children with)", "Alzheimers (Informal Caregivers of)"

Type 2 diabetes

Primary Outcomes measured in trial *

comma-separated list of primary outcomes reported in the trial

Change in objectively measured moderate-and

Secondary/other outcomes

Are there any other outcomes the intervention is expected to affect?

Changes in self-rated physical and mental health–related quality of life, physical fitness, weight, and waist circumference

Recommended "Dose" *

What do the instructions for users say on how often the app should be used?

- ( ) Approximately Daily
- (x) Approximately Weekly
- ( ) Approximately Monthly
- ( ) Approximately Yearly
- ( ) "as needed"
- ( ) Andet:

Approx. Percentage of Users (starters) still using the app as recommended after 3 months *

- (x) unknown / not evaluated
- ( ) 0-10%
- ( ) 11-20%
- ( ) 21-30%
- ( ) 31-40%
- ( ) 41-50%
- ( ) 51-60%
- ( ) 61-70%
- ( ) 71%-80%
- ( ) 81-90%
- ( ) 91-100%
- ( ) Andet:

Overall, was the app/intervention effective? *

- ( ) yes: all primary outcomes were significantly better in intervention group vs control
- ( ) partly: SOME primary outcomes were significantly better in intervention group vs control
- (x) no statistically significant difference between control and intervention
- ( ) potentially harmful: control was significantly better than intervention in one or more outcomes
- ( ) inconclusive: more research is needed
- ( ) Andet:

Article Preparation Status/Stage *

At which stage in your article preparation are you currently (at the time you fill in this form)

- ( ) not submitted yet - in early draft status
- ( ) not submitted yet - in late draft status, just before submission
- ( ) submitted to a journal but not reviewed yet
- ( ) submitted to a journal and after receiving initial reviewer comments
- (x) submitted to a journal and accepted, but not published yet
- ( ) published
- ( ) Andet:

Journal *

If you already know where you will submit this paper (or if it is already submitted), please provide the journal name (if it is not JMIR, provide the journal name under "other")

- ( ) not submitted yet / unclear where I will submit this
- ( ) Journal of Medical Internet Research (JMIR)
- (x) JMIR mHealth and UHealth
- ( ) JMIR Serious Games
- ( ) JMIR Mental Health
- ( ) JMIR Public Health
- ( ) JMIR Formative Research
- ( ) Other JMIR sister journal
- ( ) Andet:

Is this a full powered effectiveness trial or a pilot/feasibility trial? *

- ( ) Pilot/feasibility
- (x) Fully powered

Manuscript tracking number *

If this is a JMIR submission, please provide the manuscript tracking number under "other" (The ms tracking number can be found in the submission acknowledgement email, or when you login as author in JMIR. If the paper is already published in JMIR, then the ms tracking number is the four-digit number at the end of the DOI, to be found at the bottom of each published article in JMIR)

- ( ) no ms number (yet) / not (yet) submitted to / published in JMIR
- (x) Andet: 30602

## TITLE AND ABSTRACT

### 1a) TITLE: Identification as a randomized trial in the title

1a) Does your paper address CONSORT item 1a? *

I.e does the title contain the phrase "Randomized Controlled Trial"? (if not, explain the reason under "other")

- ○ yes
- ◉ Andet: The title contains "Randomized Trial", and not "Controlled" as the com

### 1a-i) Identify the mode of delivery in the title

Identify the mode of delivery. Preferably use “web-based” and/or “mobile” and/or “electronic game” in the title. Avoid ambiguous terms like “online”, “virtual”, “interactive”. Use “Internet-based” only if Intervention includes non-web-based Internet components (e.g. email), use “computer-based” or “electronic” only if offline products are used. Use “virtual” only in the context of “virtual reality” (3-D worlds). Use “online” only in the context of “online support groups”. Complement or substitute product names with broader terms for the class of products (such as “mobile” or “smart phone” instead of “iphone”), especially if the application runs on different platforms.

| | 1 | 2 | 3 | 4 | 5 | |
|---|---|---|---|---|---|---|
| subitem not at all important | ○ | ○ | ○ | ○ | ○ | essential |

Does your paper address subitem 1a-i? *

Copy and paste relevant sections from manuscript title (include quotes in quotation marks "like this" to indicate direct quotes from your manuscript), or elaborate on this item by providing additional information not in the ms, or briefly explain why the item is not applicable/relevant for your study

"Lifestyle Intervention Supported by the InterWalk Smartphone App"

1a-ii) Non-web-based components or important co-interventions in title

Mention non-web-based components or important co-interventions in title, if any (e.g., “with telephone support”).

| | 1 | 2 | 3 | 4 | 5 | |
|---|---|---|---|---|---|---|
| subitem not at all important | ○ | ○ | ○ | ○ | ○ | essential |

Does your paper address subitem 1a-ii?

Copy and paste relevant sections from manuscript title (include quotes in quotation marks "like this" to indicate direct quotes from your manuscript), or elaborate on this item by providing additional information not in the ms, or briefly explain why the item is not applicable/relevant for your study

"a Lifestyle Intervention"

1a-iii) Primary condition or target group in the title

Mention primary condition or target group in the title, if any (e.g., “for children with Type I Diabetes”) Example: A Web-based and Mobile Intervention with Telephone Support for Children with Type I Diabetes: Randomized Controlled Trial

| | 1 | 2 | 3 | 4 | 5 | |
|---|---|---|---|---|---|---|
| subitem not at all important | ○ | ○ | ○ | ○ | ○ | essential |

Does your paper address subitem 1a-iii? *

Copy and paste relevant sections from manuscript title (include quotes in quotation marks "like this" to indicate direct quotes from your manuscript), or elaborate on this item by providing additional information not in the ms, or briefly explain why the item is not applicable/relevant for your study

"Persons With Type 2 Diabetes"

1b) ABSTRACT: Structured summary of trial design, methods, results, and conclusions

NPT extension: Description of experimental treatment, comparator, care providers, centers, and blinding status.

1b-i) Key features/functionalities/components of the intervention and comparator in the METHODS section of the ABSTRACT

Mention key features/functionalities/components of the intervention and comparator in the abstract. If possible, also mention theories and principles used for designing the site. Keep in mind the needs of systematic reviewers and indexers by including important synonyms. (Note: Only report in the abstract what the main paper is reporting. If this information is missing from the main body of text, consider adding it)

| | 1 | 2 | 3 | 4 | 5 | |
|---|---|---|---|---|---|---|
| subitem not at all important | ○ | ○ | ○ | ○ | ○ | essential |

Does your paper address subitem 1b-i? *

Copy and paste relevant sections from the manuscript abstract (include quotes in quotation marks "like this" to indicate direct quotes from your manuscript), or elaborate on this item by providing additional information not in the ms, or briefly explain why the item is not applicable/relevant for your study

"(1) standard care + InterWalk app–based interval walking training (IWT; IWT group; n=140), or (2) standard care + the standard exercise program (StC group; n=74). Following 12 weeks, the IWT group was encouraged to maintain InterWalk app–based IWT (3 times per week for 30-60 minutes) and the StC group was encouraged to maintain exercise without structured support"

1b-ii) Level of human involvement in the METHODS section of the ABSTRACT

Clarify the level of human involvement in the abstract, e.g., use phrases like "fully automated" vs. "therapist/nurse/care provider/physician-assisted" (mention number and expertise of providers involved, if any). (Note: Only report in the abstract what the main paper is reporting. If this information is missing from the main body of text, consider adding it)

| | 1 | 2 | 3 | 4 | 5 | |
|---|---|---|---|---|---|---|
| subitem not at all important | ○ | ○ | ○ | ○ | ○ | essential |

Does your paper address subitem 1b-ii?

Copy and paste relevant sections from the manuscript abstract (include quotes in quotation marks "like this" to indicate direct quotes from your manuscript), or elaborate on this item by providing additional information not in the ms, or briefly explain why the item is not applicable/relevant for your study

Not relevant, as the involvement of health professionals lies in "standard care"

1b-iii) Open vs. closed, web-based (self-assessment) vs. face-to-face assessments in the METHODS section of the ABSTRACT

Mention how participants were recruited (online vs. offline), e.g., from an open access website or from a clinic or a closed online user group (closed usergroup trial), and clarify if this was a purely web-based trial, or there were face-to-face components (as part of the intervention or for assessment). Clearly say if outcomes were self-assessed through questionnaires (as common in web-based trials). Note: In traditional offline trials, an open trial (open-label trial) is a type of clinical trial in which both the researchers and participants know which treatment is being administered. To avoid confusion, use "blinded" or "unblinded" to indicated the level of blinding instead of "open", as "open" in web-based trials usually refers to "open access" (i.e. participants can self-enrol). (Note: Only report in the abstract what the main paper is reporting. If this information is missing from the main body of text, consider adding it)

| | 1 | 2 | 3 | 4 | 5 | |
|---|---|---|---|---|---|---|
| subitem not at all important | ○ | ○ | ○ | ○ | ○ | essential |

Does your paper address subitem 1b-iii?

Copy and paste relevant sections from the manuscript abstract (include quotes in quotation marks "like this" to indicate direct quotes from your manuscript), or elaborate on this item by providing additional information not in the ms, or briefly explain why the item is not applicable/relevant for your study

Not relevant, as the Objective section of the Abstract mentions that the setting of this trial is "municipality-based rehabilitation" - from which participants were recruited

1b-iv) RESULTS section in abstract must contain use data

Report number of participants enrolled/assessed in each group, the use/uptake of the intervention (e.g., attrition/adherence metrics, use over time, number of logins etc.), in addition to primary/secondary outcomes. (Note: Only report in the abstract what the main paper is reporting. If this information is missing from the main body of text, consider adding it)

| | 1 | 2 | 3 | 4 | 5 | |
|---|---|---|---|---|---|---|
| subitem not at all important | ○ | ○ | ○ | ○ | ○ | essential |

Does your paper address subitem 1b-iv?

Copy and paste relevant sections from the manuscript abstract (include quotes in quotation marks "like this" to indicate direct quotes from your manuscript), or elaborate on this item by providing additional information not in the ms, or briefly explain why the item is not applicable/relevant for your study

Number of participants is mentioned in the Methods section: "IWT group; n=140" and "StC group; n=74"; Results on primary and secondary outcomes are in the Results section of the abstract.

### 1b-v) CONCLUSIONS/DISCUSSION in abstract for negative trials

Conclusions/Discussions in abstract for negative trials: Discuss the primary outcome - if the trial is negative (primary outcome not changed), and the intervention was not used, discuss whether negative results are attributable to lack of uptake and discuss reasons. (Note: Only report in the abstract what the main paper is reporting. If this information is missing from the main body of text, consider adding it)

| | 1 | 2 | 3 | 4 | 5 | |
|---|---|---|---|---|---|---|
| subitem not at all important | ○ | ○ | ○ | ○ | ○ | essential |

### Does your paper address subitem 1b-v?

Copy and paste relevant sections from the manuscript abstract (include quotes in quotation marks "like this" to indicate direct quotes from your manuscript), or elaborate on this item by providing additional information not in the ms, or briefly explain why the item is not applicable/relevant for your study

In the abstract we conclude: "Among individuals with T2D referred to municipality-based lifestyle programs, randomization to InterWalk app–based IWT did not increase objectively measured MVPA time over 52 weeks compared with standard health care, although apparent benefits were observed for physical HRQoL."

## INTRODUCTION

### 2a) In INTRODUCTION: Scientific background and explanation of rationale

2a-i) Problem and the type of system/solution

Describe the problem and the type of system/solution that is object of the study: intended as stand-alone intervention vs. incorporated in broader health care program? Intended for a particular patient population? Goals of the intervention, e.g., being more cost-effective to other interventions, replace or complement other solutions? (Note: Details about the intervention are provided in “Methods” under 5)

| | 1 | 2 | 3 | 4 | 5 | |
|---|---|---|---|---|---|---|
| subitem not at all important | ○ | ○ | ○ | ○ | ○ | essential |

Does your paper address subitem 2a-i? *

Copy and paste relevant sections from the manuscript (include quotes in quotation marks "like this" to indicate direct quotes from your manuscript), or elaborate on this item by providing additional information not in the ms, or briefly explain why the item is not applicable/relevant for your study

The problem is described in the first paragraphs of the Introduction section. The type of system/solution that is object of the study is described in the third paragraph of the Introduction section: "The increasing implementation...". In the last paragraph of the Introduction section, it is mentioned that the solution will be implemented in a municipality-based health care setting. The population is mentioned in the second line of the Introduction section. The goal of the intervention is mentioned in the last paragraph of the Introduction section.

2a-ii) Scientific background, rationale: What is known about the (type of) system

Scientific background, rationale: What is known about the (type of) system that is the object of the study (be sure to discuss the use of similar systems for other conditions/diagnoses, if appropiate), motivation for the study, i.e. what are the reasons for and what is the context for this specific study, from which stakeholder viewpoint is the study performed, potential impact of findings [2]. Briefly justify the choice of the comparator.

| | 1 | 2 | 3 | 4 | 5 | |
|---|---|---|---|---|---|---|
| subitem not at all important | ○ | ○ | ○ | ○ | ○ | essential |

Does your paper address subitem 2a-ii? *

Copy and paste relevant sections from the manuscript (include quotes in quotation marks "like this" to indicate direct quotes from your manuscript), or elaborate on this item by providing additional information not in the ms, or briefly explain why the item is not applicable/relevant for your study

The scientific background and rationale are discussed in the second and third paragraph of the Introduction section

2b) In INTRODUCTION: Specific objectives or hypotheses

Does your paper address CONSORT subitem 2b? *

Copy and paste relevant sections from the manuscript (include quotes in quotation marks "like this" to indicate direct quotes from your manuscript), or elaborate on this item by providing additional information not in the ms, or briefly explain why the item is not applicable/relevant for your study

The hypothesis is stated in the last paragraph of the Introduction section: "the hypothesis that InterWalk app–based IWT implemented in a municipality-based health care setting is superior in increasing MVPA across 52 weeks compared with standard care among individuals with T2D"

METHODS

3a) Description of trial design (such as parallel, factorial) including allocation ratio

Does your paper address CONSORT subitem 3a? *

Copy and paste relevant sections from the manuscript (include quotes in quotation marks "like this" to indicate direct quotes from your manuscript), or elaborate on this item by providing additional information not in the ms, or briefly explain why the item is not applicable/relevant for your study

The study design is described in the "Study Design" subsection of the Methods section. The allocation ratio is mentioned in the beginning of the "Randomization and Blinding" subsection of the Methods section

3b) Important changes to methods after trial commencement (such as eligibility criteria), with reasons

Does your paper address CONSORT subitem 3b? *

Copy and paste relevant sections from the manuscript (include quotes in quotation marks "like this" to indicate direct quotes from your manuscript), or elaborate on this item by providing additional information not in the ms, or briefly explain why the item is not applicable/relevant for your study

Protocol changes of secondary outcomes are listed on page 8 of the Statistical Analysis Plan (Multimedia Appendix 1)

3b-i) Bug fixes, Downtimes, Content Changes

Bug fixes, Downtimes, Content Changes: ehealth systems are often dynamic systems. A description of changes to methods therefore also includes important changes made on the intervention or comparator during the trial (e.g., major bug fixes or changes in the functionality or content) (5-iii) and other “unexpected events” that may have influenced study design such as staff changes, system failures/downtimes, etc. [2].

| | 1 | 2 | 3 | 4 | 5 | |
|---|---|---|---|---|---|---|
| subitem not at all important | ○ | ○ | ○ | ○ | ○ | essential |

Does your paper address subitem 3b-i?

Copy and paste relevant sections from the manuscript (include quotes in quotation marks "like this" to indicate direct quotes from your manuscript), or elaborate on this item by providing additional information not in the ms, or briefly explain why the item is not applicable/relevant for your study

Downtime of the app is described in the Methods section: "a period of InterWalk app malfunctions connected to a major restructuring of the iOS (version 9; available during the period from September 16 to October 21, 2016)"

4a) Eligibility criteria for participants

Does your paper address CONSORT subitem 4a? *

Copy and paste relevant sections from the manuscript (include quotes in quotation marks "like this" to indicate direct quotes from your manuscript), or elaborate on this item by providing additional information not in the ms, or briefly explain why the item is not applicable/relevant for your study

Eligibility criteria are described in the "Participants and Eligibility" subsection of the Methods section

4a-i) Computer / Internet literacy

Computer / Internet literacy is often an implicit “de facto” eligibility criterion - this should be explicitly clarified.

| | 1 | 2 | 3 | 4 | 5 | |
|---|---|---|---|---|---|---|
| subitem not at all important | ○ | ○ | ○ | ○ | ○ | essential |

Does your paper address subitem 4a-i?

Copy and paste relevant sections from the manuscript (include quotes in quotation marks "like this" to indicate direct quotes from your manuscript), or elaborate on this item by providing additional information not in the ms, or briefly explain why the item is not applicable/relevant for your study

Computer/Internet literacy is not described as an eligibility criterion as this was not considered when recruiting for the trial

4a-ii) Open vs. closed, web-based vs. face-to-face assessments:

Open vs. closed, web-based vs. face-to-face assessments: Mention how participants were recruited (online vs. offline), e.g., from an open access website or from a clinic, and clarify if this was a purely web-based trial, or there were face-to-face components (as part of the intervention or for assessment), i.e., to what degree got the study team to know the participant. In online-only trials, clarify if participants were quasi-anonymous and whether having multiple identities was possible or whether technical or logistical measures (e.g., cookies, email confirmation, phone calls) were used to detect/prevent these.

| | 1 | 2 | 3 | 4 | 5 | |
|---|---|---|---|---|---|---|
| subitem not at all important | ○ | ○ | ○ | ○ | ○ | essential |

Does your paper address subitem 4a-ii? *

Copy and paste relevant sections from the manuscript (include quotes in quotation marks "like this" to indicate direct quotes from your manuscript), or elaborate on this item by providing additional information not in the ms, or briefly explain why the item is not applicable/relevant for your study

Recruitment is described as: "All individuals who met for an appointment at the participating health promotion centers or hospital were screened through medical records and at a screening interview with a health professional at the center or hospital"; The nature of the interventions including face-to-face components are described in the "Baseline to 12-Week Follow-up" and "From 12-Week to 52-Week Follow-up" subsections of the Methods section

4a-iii) Information giving during recruitment

Information given during recruitment. Specify how participants were briefed for recruitment and in the informed consent procedures (e.g., publish the informed consent documentation as appendix, see also item X26), as this information may have an effect on user self-selection, user expectation and may also bias results.

| | 1 | 2 | 3 | 4 | 5 | |
|---|---|---|---|---|---|---|
| subitem not at all important | ○ | ○ | ○ | ○ | ○ | essential |

Does your paper address subitem 4a-iii?

Copy and paste relevant sections from the manuscript (include quotes in quotation marks "like this" to indicate direct quotes from your manuscript), or elaborate on this item by providing additional information not in the ms, or briefly explain why the item is not applicable/relevant for your study

"All participants provided oral and written informed consent prior to commencing any study procedures"

4b) Settings and locations where the data were collected

Does your paper address CONSORT subitem 4b? *

Copy and paste relevant sections from the manuscript (include quotes in quotation marks "like this" to indicate direct quotes from your manuscript), or elaborate on this item by providing additional information not in the ms, or briefly explain why the item is not applicable/relevant for your study

"The data were collected at the participating health promotion centers (Amager, Vanløse, Østerbro, Vesterbro in the Municipality of Copenhagen, Denmark; and Municipality of Guldborgsund, Denmark) and a hospital (Bornholm, Denmark)."

4b-i) Report if outcomes were (self-)assessed through online questionnaires

Clearly report if outcomes were (self-)assessed through online questionnaires (as common in web-based trials) or otherwise.

| | 1 | 2 | 3 | 4 | 5 | |
|---|---|---|---|---|---|---|
| subitem not at all important | ○ | ○ | ○ | ○ | ○ | essential |

Does your paper address subitem 4b-i? *

Copy and paste relevant sections from the manuscript (include quotes in quotation marks "like this" to indicate direct quotes from your manuscript), or elaborate on this item by providing additional information not in the ms, or briefly explain why the item is not applicable/relevant for your study

In the Outcomes subsection of the Methods section, we refer to the previously published protocol: "Primary and key secondary outcome measurements are described in further detail in the published protocol [23]." The published protocol contains a thorough description of data management in the Data management and quality control subsection of the Methods and analysis section, including: "All data are entered in an secure sockets layer (SSL–secured, online-based database developed by the Danish Stakeholder Help2Run (CVR: 34801088). [...] Data on demography, measurements, tests and questionnaires are entered directly in the database"

4b-ii) Report how institutional affiliations are displayed

Report how institutional affiliations are displayed to potential participants [on ehealth media], as affiliations with prestigious hospitals or universities may affect volunteer rates, use, and reactions with regards to an intervention.(Not a required item – describe only if this may bias results)

| | 1 | 2 | 3 | 4 | 5 | |
|---|---|---|---|---|---|---|
| subitem not at all important | ○ | ○ | ○ | ○ | ○ | essential |

Does your paper address subitem 4b-ii?

Copy and paste relevant sections from the manuscript (include quotes in quotation marks "like this" to indicate direct quotes from your manuscript), or elaborate on this item by providing additional information not in the ms, or briefly explain why the item is not applicable/relevant for your study

Not applicable...........

5) The interventions for each group with sufficient details to allow replication, including how and when they were actually administered

5-i) Mention names, credential, affiliations of the developers, sponsors, and owners

Mention names, credential, affiliations of the developers, sponsors, and owners [6] (if authors/evaluators are owners or developer of the software, this needs to be declared in a “Conflict of interest” section or mentioned elsewhere in the manuscript).

| | 1 | 2 | 3 | 4 | 5 | |
|---|---|---|---|---|---|---|
| subitem not at all important | ○ | ○ | ○ | ○ | ○ | essential |

Does your paper address subitem 5-i?

Copy and paste relevant sections from the manuscript (include quotes in quotation marks "like this" to indicate direct quotes from your manuscript), or elaborate on this item by providing additional information not in the ms, or briefly explain why the item is not applicable/relevant for your study

We refer to a paper previously published in the "The InterWalk App" subsection of the Methods section (Reference #20)

5-ii) Describe the history/development process

Describe the history/development process of the application and previous formative evaluations (e.g., focus groups, usability testing), as these will have an impact on adoption/use rates and help with interpreting results.

| | 1 | 2 | 3 | 4 | 5 | |
|---|---|---|---|---|---|---|
| subitem not at all important | ○ | ○ | ○ | ○ | ○ | essential |

Does your paper address subitem 5-ii?

Copy and paste relevant sections from the manuscript (include quotes in quotation marks "like this" to indicate direct quotes from your manuscript), or elaborate on this item by providing additional information not in the ms, or briefly explain why the item is not applicable/relevant for your study

This is described in detail in the preveiously published paper (Reference #20) and briefly in the "The InterWalk App" subsection of the Methods section

5-iii) Revisions and updating

Revisions and updating. Clearly mention the date and/or version number of the application/intervention (and comparator, if applicable) evaluated, or describe whether the intervention underwent major changes during the evaluation process, or whether the development and/or content was “frozen” during the trial. Describe dynamic components such as news feeds or changing content which may have an impact on the replicability of the intervention (for unexpected events see item 3b).

| | 1 | 2 | 3 | 4 | 5 | |
|---|---|---|---|---|---|---|
| subitem not at all important | ○ | ○ | ○ | ○ | ○ | essential |

Does your paper address subitem 5-iii?

Copy and paste relevant sections from the manuscript (include quotes in quotation marks "like this" to indicate direct quotes from your manuscript), or elaborate on this item by providing additional information not in the ms, or briefly explain why the item is not applicable/relevant for your study

"... InterWalk app malfunctions connected to a major restructuring of the iOS (version 9; available during the period from September 16 to October 21, 2016)"

5-iv) Quality assurance methods

Provide information on quality assurance methods to ensure accuracy and quality of information provided [1], if applicable.

| | 1 | 2 | 3 | 4 | 5 | |
|---|---|---|---|---|---|---|
| subitem not at all important | ○ | ○ | ○ | ○ | ○ | essential |

Does your paper address subitem 5-iv?

Copy and paste relevant sections from the manuscript (include quotes in quotation marks "like this" to indicate direct quotes from your manuscript), or elaborate on this item by providing additional information not in the ms, or briefly explain why the item is not applicable/relevant for your study

Not applicable...........

5-v) Ensure replicability by publishing the source code, and/or providing screenshots/screen-capture video, and/or providing flowcharts of the algorithms used

Ensure replicability by publishing the source code, and/or providing screenshots/screen-capture video, and/or providing flowcharts of the algorithms used. Replicability (i.e., other researchers should in principle be able to replicate the study) is a hallmark of scientific reporting.

| | 1 | 2 | 3 | 4 | 5 | |
|---|---|---|---|---|---|---|
| subitem not at all important | ○ | ○ | ○ | ○ | ○ | essential |

Does your paper address subitem 5-v?

Copy and paste relevant sections from the manuscript (include quotes in quotation marks "like this" to indicate direct quotes from your manuscript), or elaborate on this item by providing additional information not in the ms, or briefly explain why the item is not applicable/relevant for your study

Regarding the InterWalk app: "The full details have been described in detail elsewhere [20]", including screenshots, etc.

5-vi) Digital preservation

Digital preservation: Provide the URL of the application, but as the intervention is likely to change or disappear over the course of the years; also make sure the intervention is archived (Internet Archive, webcitation.org, and/or publishing the source code or screenshots/videos alongside the article). As pages behind login screens cannot be archived, consider creating demo pages which are accessible without login.

| | 1 | 2 | 3 | 4 | 5 | |
|---|---|---|---|---|---|---|
| subitem not at all important | ○ | ○ | ○ | ○ | ○ | essential |

Does your paper address subitem 5-vi?

Copy and paste relevant sections from the manuscript (include quotes in quotation marks "like this" to indicate direct quotes from your manuscript), or elaborate on this item by providing additional information not in the ms, or briefly explain why the item is not applicable/relevant for your study

We refer to a paper describing the development and design of the InterWalk app in detail (Reference #20)

5-vii) Access

Access: Describe how participants accessed the application, in what setting/context, if they had to pay (or were paid) or not, whether they had to be a member of specific group. If known, describe how participants obtained "access to the platform and Internet" [1]. To ensure access for editors/reviewers/readers, consider to provide a "backdoor" login account or demo mode for reviewers/readers to explore the application (also important for archiving purposes, see vi).

| | 1 | 2 | 3 | 4 | 5 | |
|---|---|---|---|---|---|---|
| subitem not at all important | ○ | ○ | ○ | ○ | ○ | essential |

Does your paper address subitem 5-vii? *

Copy and paste relevant sections from the manuscript (include quotes in quotation marks "like this" to indicate direct quotes from your manuscript), or elaborate on this item by providing additional information not in the ms, or briefly explain why the item is not applicable/relevant for your study

"The IWT group was prescribed InterWalk app–based IWT (30-60 minutes per session, 3 sessions per week for 12 weeks; for an extended description, see below) instead of the standard exercise program. During the 12-week exercise program, IWT was group based and 2 of the 3 sessions were supervised."

5-viii) Mode of delivery, features/functionalities/components of the intervention and comparator, and the theoretical framework

Describe mode of delivery, features/functionalities/components of the intervention and comparator, and the theoretical framework [6] used to design them (instructional strategy [1], behaviour change techniques, persuasive features, etc., see e.g., [7, 8] for terminology). This includes an in-depth description of the content (including where it is coming from and who developed it) [1]," whether [and how] it is tailored to individual circumstances and allows users to track their progress and receive feedback" [6]. This also includes a description of communication delivery channels and – if computer-mediated communication is a component – whether communication was synchronous or asynchronous [6]. It also includes information on presentation strategies [1], including page design principles, average amount of text on pages, presence of hyperlinks to other resources, etc. [1].

| | 1 | 2 | 3 | 4 | 5 | |
|---|---|---|---|---|---|---|
| subitem not at all important | ○ | ○ | ○ | ○ | ○ | essential |

Does your paper address subitem 5-viii? *

Copy and paste relevant sections from the manuscript (include quotes in quotation marks "like this" to indicate direct quotes from your manuscript), or elaborate on this item by providing additional information not in the ms, or briefly explain why the item is not applicable/relevant for your study

This is described in the Interventions subsection of the Methods section

5-ix) Describe use parameters

Describe use parameters (e.g., intended "doses" and optimal timing for use). Clarify what instructions or recommendations were given to the user, e.g., regarding timing, frequency, heaviness of use, if any, or was the intervention used ad libitum.

| | 1 | 2 | 3 | 4 | 5 | |
|---|---|---|---|---|---|---|
| subitem not at all important | ○ | ○ | ○ | ○ | ○ | essential |

Does your paper address subitem 5-ix?

Copy and paste relevant sections from the manuscript (include quotes in quotation marks "like this" to indicate direct quotes from your manuscript), or elaborate on this item by providing additional information not in the ms, or briefly explain why the item is not applicable/relevant for your study

"The IWT group was prescribed InterWalk app–based IWT (30-60 minutes per session, 3 sessions per week for 12 weeks; for an extended description, see below) instead of the standard exercise program." and "The participants allocated to the InterWalk app–based intervention from baseline to 12-week follow-up were either allocated to be encouraged to maintain InterWalk app–based IWT (3 times per week for 30-60 minutes; IWTonly) until 52-week follow-up or was, for explorative purposes, allocated to additional motivational support following the 12-week program and until the 52-week follow-up (IWTsupport)."

5-x) Clarify the level of human involvement

Clarify the level of human involvement (care providers or health professionals, also technical assistance) in the e-intervention or as co-intervention (detail number and expertise of professionals involved, if any, as well as “type of assistance offered, the timing and frequency of the support, how it is initiated, and the medium by which the assistance is delivered”. It may be necessary to distinguish between the level of human involvement required for the trial, and the level of human involvement required for a routine application outside of a RCT setting (discuss under item 21 – generalizability).

| | 1 | 2 | 3 | 4 | 5 | |
|---|---|---|---|---|---|---|
| subitem not at all important | ○ | ○ | ○ | ○ | ○ | essential |

Does your paper address subitem 5-x?

Copy and paste relevant sections from the manuscript (include quotes in quotation marks "like this" to indicate direct quotes from your manuscript), or elaborate on this item by providing additional information not in the ms, or briefly explain why the item is not applicable/relevant for your study

"During the 12-week exercise program, IWT was group based and 2 of the 3 sessions were supervised." and for the IWTsupport subgroup "The motivational support included (1) individual motivational interviews with individual goal setting performed by the health care professionals, (2) InterWalk-based IWT with voluntary ambassadors affiliated with the Danish Diabetes Association (a Danish not-for-profit patient organization) once per week, ..."

5-xi) Report any prompts/reminders used

Report any prompts/reminders used: Clarify if there were prompts (letters, emails, phone calls, SMS) to use the application, what triggered them, frequency etc. It may be necessary to distinguish between the level of prompts/reminders required for the trial, and the level of prompts/reminders for a routine application outside of a RCT setting (discuss under item 21 – generalizability).

| | 1 | 2 | 3 | 4 | 5 | |
|---|---|---|---|---|---|---|
| subitem not at all important | ○ | ○ | ○ | ○ | ○ | essential |

Does your paper address subitem 5-xi? *

Copy and paste relevant sections from the manuscript (include quotes in quotation marks "like this" to indicate direct quotes from your manuscript), or elaborate on this item by providing additional information not in the ms, or briefly explain why the item is not applicable/relevant for your study

For the IWTsupport subgroup "facilitation of high adherence to IWT was based on automated feedback to self-reported adherence to IWT using weekly bidirectional SMS text message surveys [24]. An automatic survey was sent during the afternoon on Sundays and inquired about the frequency of IWT during the past week"

5-xii) Describe any co-interventions (incl. training/support)

Describe any co-interventions (incl. training/support): Clearly state any interventions that are provided in addition to the targeted eHealth intervention, as ehealth intervention may not be designed as stand-alone intervention. This includes training sessions and support [1]. It may be necessary to distinguish between the level of training required for the trial, and the level of training for a routine application outside of a RCT setting (discuss under item 21 – generalizability.

| | 1 | 2 | 3 | 4 | 5 | |
|---|---|---|---|---|---|---|
| subitem not at all important | ○ | ○ | ○ | ○ | ○ | essential |

Does your paper address subitem 5-xii? *

Copy and paste relevant sections from the manuscript (include quotes in quotation marks "like this" to indicate direct quotes from your manuscript), or elaborate on this item by providing additional information not in the ms, or briefly explain why the item is not applicable/relevant for your study

"As part of normal practice, lifestyle interventions may be prescribed to individuals with T2D by their general practitioner by referral to municipality-based lifestyle programs [26]. This program entails a combination of exercise, diabetes education on self-management, smoking cessation courses, and diet counseling. A patient might receive all or any combination of these components, depending on the specific need of the patient. The decision is based on a dialog between the patient and the health care provider upon initiation of the program.
Both the IWT and StC groups underwent 12 weeks of standard municipality-based health care [23,26]. They both received an exercise program during the initial 12 weeks." and for the IWTsupport subgroup "The motivational support included (1) individual motivational interviews with individual goal setting performed by the health care professionals, (2) InterWalk-based IWT with voluntary ambassadors affiliated with the Danish Diabetes Association (a Danish not-for-profit patient organization) once per week, and (3) SMS text message support (once per week)."

6a) Completely defined pre-specified primary and secondary outcome measures, including how and when they were assessed

Does your paper address CONSORT subitem 6a? *

Copy and paste relevant sections from the manuscript (include quotes in quotation marks "like this" to indicate direct quotes from your manuscript), or elaborate on this item by providing additional information not in the ms, or briefly explain why the item is not applicable/relevant for your study

Primary and secondary outcomes measures are described in the "Outcomes" subsection of the Methods section

### 6a-i) Online questionnaires: describe if they were validated for online use and apply CHERRIES items to describe how the questionnaires were designed/deployed

If outcomes were obtained through online questionnaires, describe if they were validated for online use and apply CHERRIES items to describe how the questionnaires were designed/deployed [9].

| | 1 | 2 | 3 | 4 | 5 | |
|---|---|---|---|---|---|---|
| subitem not at all important | ○ | ○ | ○ | ○ | ○ | essential |

### Does your paper address subitem 6a-i?

Copy and paste relevant sections from manuscript text

Not applicable...........

### 6a-ii) Describe whether and how “use” (including intensity of use/dosage) was defined/measured/monitored

Describe whether and how “use” (including intensity of use/dosage) was defined/measured/monitored (logins, logfile analysis, etc.). Use/adoption metrics are important process outcomes that should be reported in any ehealth trial.

| | 1 | 2 | 3 | 4 | 5 | |
|---|---|---|---|---|---|---|
| subitem not at all important | ○ | ○ | ○ | ○ | ○ | essential |

Does your paper address subitem 6a-ii?

Copy and paste relevant sections from manuscript text

Monitoring: "An automatic survey was sent during the afternoon on Sundays and inquired about the frequency of IWT during the past week (1="I have walked more than 3 times/week", 2="I have walked 3 times/week", 3="I have walked 2 times/week", 4="I have walked 1 times/week", and 5="I did not walk"). If no answer was received, a reminder was sent within 24 hours. If no answer was received following the reminder or the participant answered, "I did not walk," the health care professional was instructed to reach the participant by phone using a semistructured approach. The semistructured interview guide consisted of 2 overall questions: (1) Do you experience barriers toward IWT? If yes, which ones? (2) How can I help you to overcome these barriers? If the participant indicated walking 1-2 times/week, an automated SMS text message was sent encouraging the participant to walk more."
Measurement: "Following an IWT session, the objectively measured adherence (intensity, duration, and frequency) to IWT sessions was calculated based on the electronically logged information from the InterWalk app. Data from the InterWalk app were continuously uploaded to a central and secure server throughout the duration of the trial. Self-reported adherence to IWT was calculated based on data from the SMS text message survey, including frequency of weekly use of the InterWalk app and reasons for not using the InterWalk app [24]."

6a-iii) Describe whether, how, and when qualitative feedback from participants was obtained

Describe whether, how, and when qualitative feedback from participants was obtained (e.g., through emails, feedback forms, interviews, focus groups).

| | 1 | 2 | 3 | 4 | 5 | |
|---|---|---|---|---|---|---|
| subitem not at all important | ○ | ○ | ○ | ○ | ○ | essential |

Does your paper address subitem 6a-iii?

Copy and paste relevant sections from manuscript text

The only available qualitative feedback was what was obtained through the motivational interviews with the IWTsupport subgroup. This is reported in Multimedia Appendix 5.

6b) Any changes to trial outcomes after the trial commenced, with reasons

Does your paper address CONSORT subitem 6b? *

Copy and paste relevant sections from the manuscript (include quotes in quotation marks "like this" to indicate direct quotes from your manuscript), or elaborate on this item by providing additional information not in the ms, or briefly explain why the item is not applicable/relevant for your study

Changes to trial outcomes are described in the Statistical Analysis Plan (Multimedia Appendix 1)

7a) How sample size was determined

NPT: When applicable, details of whether and how the clustering by care provides or centers was addressed

7a-i) Describe whether and how expected attrition was taken into account when calculating the sample size

Describe whether and how expected attrition was taken into account when calculating the sample size.

| | 1 | 2 | 3 | 4 | 5 | |
|---|---|---|---|---|---|---|
| subitem not at all important | ○ | ○ | ○ | ○ | ○ | essential |

Does your paper address subitem 7a-i?

Copy and paste relevant sections from manuscript title (include quotes in quotation marks "like this" to indicate direct quotes from your manuscript), or elaborate on this item by providing additional information not in the ms, or briefly explain why the item is not applicable/relevant for your study

"Allowing for 30% attrition, 272 participants (91 in the StC group and 181 in the IWT group) should be recruited."

7b) When applicable, explanation of any interim analyses and stopping guidelines

Does your paper address CONSORT subitem 7b? *

Copy and paste relevant sections from the manuscript (include quotes in quotation marks "like this" to indicate direct quotes from your manuscript), or elaborate on this item by providing additional information not in the ms, or briefly explain why the item is not applicable/relevant for your study

"The intervention settings enabled recruitment until December 15, 2016, and thus the sample size would include 272 participants or truncated at the number of participants included at the end of the recruitment period—whichever was reached first"

8a) Method used to generate the random allocation sequence
NPT: When applicable, how care providers were allocated to each trial group

Does your paper address CONSORT subitem 8a? *

Copy and paste relevant sections from the manuscript (include quotes in quotation marks "like this" to indicate direct quotes from your manuscript), or elaborate on this item by providing additional information not in the ms, or briefly explain why the item is not applicable/relevant for your study

"The allocation sequence was generated through a standardized computer program by an independent statistician (RC)"

8b) Type of randomisation; details of any restriction (such as blocking and block size)

Does your paper address CONSORT subitem 8b? *

Copy and paste relevant sections from the manuscript (include quotes in quotation marks "like this" to indicate direct quotes from your manuscript), or elaborate on this item by providing additional information not in the ms, or briefly explain why the item is not applicable/relevant for your study

"Participants were randomized using random permuted blocks stratified by sex (2 levels) and center (6 levels)."

9) Mechanism used to implement the random allocation sequence (such as sequentially numbered containers), describing any steps taken to conceal the sequence until interventions were assigned

Does your paper address CONSORT subitem 9? *

Copy and paste relevant sections from the manuscript (include quotes in quotation marks "like this" to indicate direct quotes from your manuscript), or elaborate on this item by providing additional information not in the ms, or briefly explain why the item is not applicable/relevant for your study

"... stored on a password-protected computer by an independent researcher (RN) who was not involved in any study procedures. Following the completion of all baseline measurements, the independent researcher was contacted and performed allocation (to StC or IWTonly or IWTsupport). The respective group allocation for the initial 12 weeks (StC or IWT) was subsequently returned by email to the health professional who informed the participant about the allocation by telephone call. Information about IWTonly or IWTsupport was not disclosed by the independent researcher to the health professionals until week 12. Following the 12-week intermediate assessment, the independent researcher was contacted and the allocation into IWTonly or IWTsupport was returned by email to the health professional who informed the participant about the allocation by telephone call."

10) Who generated the random allocation sequence, who enrolled participants, and who assigned participants to interventions

Does your paper address CONSORT subitem 10? *

Copy and paste relevant sections from the manuscript (include quotes in quotation marks "like this" to indicate direct quotes from your manuscript), or elaborate on this item by providing additional information not in the ms, or briefly explain why the item is not applicable/relevant for your study

"The allocation sequence was generated through a standardized computer program by an independent statistician (RC)"; "All individuals who met for an appointment at the participating health promotion centers or hospital were screened through medical records and at a screening interview with a health professional at the center or hospital."; and "... stored on a password-protected computer by an independent researcher (RN) who was not involved in any study procedures. Following the completion of all baseline measurements, the independent researcher was contacted and performed allocation (to StC or IWTonly or IWTsupport)."

11a) If done, who was blinded after assignment to interventions (for example, participants, care providers, those assessing outcomes) and how

NPT: Whether or not administering co-interventions were blinded to group assignment

11a-i) Specify who was blinded, and who wasn't

Specify who was blinded, and who wasn't. Usually, in web-based trials it is not possible to blind the participants [1, 3] (this should be clearly acknowledged), but it may be possible to blind outcome assessors, those doing data analysis or those administering co-interventions (if any).

| | 1 | 2 | 3 | 4 | 5 | |
|---|---|---|---|---|---|---|
| subitem not at all important | O | O | O | O | O | essential |

Does your paper address subitem 11a-i? *

Copy and paste relevant sections from the manuscript (include quotes in quotation marks "like this" to indicate direct quotes from your manuscript), or elaborate on this item by providing additional information not in the ms, or briefly explain why the item is not applicable/relevant for your study

"The health professionals carried through the data collection and intervention, and thus, were only blinded to the primary outcome."

11a-ii) Discuss e.g., whether participants knew which intervention was the "intervention of interest" and which one was the "comparator"

Informed consent procedures (4a-ii) can create biases and certain expectations - discuss e.g., whether participants knew which intervention was the "intervention of interest" and which one was the "comparator".

| | 1 | 2 | 3 | 4 | 5 | |
|---|---|---|---|---|---|---|
| subitem not at all important | O | O | O | O | O | essential |

### Does your paper address subitem 11a-ii?

Copy and paste relevant sections from the manuscript (include quotes in quotation marks "like this" to indicate direct quotes from your manuscript), or elaborate on this item by providing additional information not in the ms, or briefly explain why the item is not applicable/relevant for your study

Not applicable. As described in the Interventions subsection of the Methods section, InterWalk app-based interval walking training replaced a standard municipality-based exercise program for the IWT group.

### 11b) If relevant, description of the similarity of interventions

(this item is usually not relevant for ehealth trials as it refers to similarity of a placebo or sham intervention to a active medication/intervention)

### Does your paper address CONSORT subitem 11b? *

Copy and paste relevant sections from the manuscript (include quotes in quotation marks "like this" to indicate direct quotes from your manuscript), or elaborate on this item by providing additional information not in the ms, or briefly explain why the item is not applicable/relevant for your study

"Both the IWT and StC groups underwent 12 weeks of standard municipality-based health care [23,26]. They both received an exercise program during the initial 12 weeks."

### 12a) Statistical methods used to compare groups for primary and secondary outcomes

NPT: When applicable, details of whether and how the clustering by care providers or centers was addressed

Does your paper address CONSORT subitem 12a? *

Copy and paste relevant sections from the manuscript (include quotes in quotation marks "like this" to indicate direct quotes from your manuscript), or elaborate on this item by providing additional information not in the ms, or briefly explain why the item is not applicable/relevant for your study

Statistical methods are described in the "Statistical Methods" subsection of the Methods section

12a-i) Imputation techniques to deal with attrition / missing values

Imputation techniques to deal with attrition / missing values: Not all participants will use the intervention/comparator as intended and attrition is typically high in ehealth trials. Specify how participants who did not use the application or dropped out from the trial were treated in the statistical analysis (a complete case analysis is strongly discouraged, and simple imputation techniques such as LOCF may also be problematic [4]).

| | 1 | 2 | 3 | 4 | 5 | |
|---|---|---|---|---|---|---|
| subitem not at all important | ○ | ○ | ○ | ○ | ○ | essential |

Does your paper address subitem 12a-i? *

Copy and paste relevant sections from the manuscript (include quotes in quotation marks "like this" to indicate direct quotes from your manuscript), or elaborate on this item by providing additional information not in the ms, or briefly explain why the item is not applicable/relevant for your study

As described in the "Statistical Methods" subsection, we analyzed data using repeated-measures mixed linear models under the assumption that data were missing at random. In a sensitivity analysis of the primary outcome we implemented baseline observation carried forward, which supported the primary analysis.

12b) Methods for additional analyses, such as subgroup analyses and adjusted analyses

Does your paper address CONSORT subitem 12b? *

Copy and paste relevant sections from the manuscript (include quotes in quotation marks "like this" to indicate direct quotes from your manuscript), or elaborate on this item by providing additional information not in the ms, or briefly explain why the item is not applicable/relevant for your study

Sensitivity analyses and subgroup analyses are described in the "Statistical Methods" subsection of the Methods section

X26) REB/IRB Approval and Ethical Considerations [recommended as subheading under "Methods"] (not a CONSORT item)

X26-i) Comment on ethics committee approval

| | 1 | 2 | 3 | 4 | 5 | |
|---|---|---|---|---|---|---|
| subitem not at all important | ○ | ○ | ○ | ○ | ○ | essential |

Does your paper address subitem X26-i?

Copy and paste relevant sections from the manuscript (include quotes in quotation marks "like this" to indicate direct quotes from your manuscript), or elaborate on this item by providing additional information not in the ms, or briefly explain why the item is not applicable/relevant for your study

"The study was approved by the scientific ethical committee of the Capital Region of Denmark (H-1-2014-074)"

x26-ii) Outline informed consent procedures

Outline informed consent procedures e.g., if consent was obtained offline or online (how? Checkbox, etc.?), and what information was provided (see 4a-ii). See [6] for some items to be included in informed consent documents.

| | 1 | 2 | 3 | 4 | 5 | |
|---|---|---|---|---|---|---|
| subitem not at all important | ○ | ○ | ○ | ○ | ○ | essential |

Does your paper address subitem X26-ii?

Copy and paste relevant sections from the manuscript (include quotes in quotation marks "like this" to indicate direct quotes from your manuscript), or elaborate on this item by providing additional information not in the ms, or briefly explain why the item is not applicable/relevant for your study

"All participants provided oral and written informed consent prior to commencing any study procedures"

X26-iii) Safety and security procedures

Safety and security procedures, incl. privacy considerations, and any steps taken to reduce the likelihood or detection of harm (e.g., education and training, availability of a hotline)

| | 1 | 2 | 3 | 4 | 5 | |
|---|---|---|---|---|---|---|
| subitem not at all important | ○ | ○ | ○ | ○ | ○ | essential |

Does your paper address subitem X26-iii?

Copy and paste relevant sections from the manuscript (include quotes in quotation marks "like this" to indicate direct quotes from your manuscript), or elaborate on this item by providing additional information not in the ms, or briefly explain why the item is not applicable/relevant for your study

The intervention was implemented in a municipality-based health care setting and thus health professionals was involved in participants' education and training, meeting with them 2 times per week for 12 weeks. This is described in the "Baseline to 12-week follow-up" subsection of the Methods section

RESULTS

13a) For each group, the numbers of participants who were randomly assigned, received intended treatment, and were analysed for the primary outcome
NPT: The number of care providers or centers performing the intervention in each group and the number of patients treated by each care provider in each center

Does your paper address CONSORT subitem 13a? *

Copy and paste relevant sections from the manuscript (include quotes in quotation marks "like this" to indicate direct quotes from your manuscript), or elaborate on this item by providing additional information not in the ms, or briefly explain why the item is not applicable/relevant for your study

"Of the 214 participants enrolled in the study, 74 participants were allocated to the StC group and 140 participants to the IWT group" and Figure 1

13b) For each group, losses and exclusions after randomisation, together with reasons

Does your paper address CONSORT subitem 13b? (NOTE: Preferably, this is shown in a CONSORT flow diagram) *

Copy and paste relevant sections from the manuscript (include quotes in quotation marks "like this" to indicate direct quotes from your manuscript), or elaborate on this item by providing additional information not in the ms, or briefly explain why the item is not applicable/relevant for your study

Lost to follow-up are depicted in Figure 1

13b-i) Attrition diagram

Strongly recommended: An attrition diagram (e.g., proportion of participants still logging in or using the intervention/comparator in each group plotted over time, similar to a survival curve) or other figures or tables demonstrating usage/dose/engagement.

| | 1 | 2 | 3 | 4 | 5 | |
|---|---|---|---|---|---|---|
| subitem not at all important | ○ | ○ | ○ | ○ | ○ | essential |

Does your paper address subitem 13b-i?

Copy and paste relevant sections from the manuscript or cite the figure number if applicable (include quotes in quotation marks "like this" to indicate direct quotes from your manuscript), or elaborate on this item by providing additional information not in the ms, or briefly explain why the item is not applicable/relevant for your study

Adherence to the IWT intervention is presented in Multimedia Appendix 4 and attrition is depicted in Figure 1

14a) Dates defining the periods of recruitment and follow-up

Does your paper address CONSORT subitem 14a? *

Copy and paste relevant sections from the manuscript (include quotes in quotation marks "like this" to indicate direct quotes from your manuscript), or elaborate on this item by providing additional information not in the ms, or briefly explain why the item is not applicable/relevant for your study

"Between January 2015 and December 2016, 762 individuals were screened for inclusion" and "A total of 130 participants completed the 52-week follow-up, which was finalized in December 2017."

14a-i) Indicate if critical “secular events” fell into the study period

Indicate if critical “secular events” fell into the study period, e.g., significant changes in Internet resources available or “changes in computer hardware or Internet delivery resources”

| | 1 | 2 | 3 | 4 | 5 | |
|---|---|---|---|---|---|---|
| subitem not at all important | ○ | ○ | ○ | ○ | ○ | essential |

Does your paper address subitem 14a-i?

Copy and paste relevant sections from the manuscript (include quotes in quotation marks "like this" to indicate direct quotes from your manuscript), or elaborate on this item by providing additional information not in the ms, or briefly explain why the item is not applicable/relevant for your study

"a period of InterWalk app malfunctions connected to a major restructuring of the iOS (version 9; available during the period from September 16 to October 21, 2016)"

14b) Why the trial ended or was stopped (early)

Does your paper address CONSORT subitem 14b? *

Copy and paste relevant sections from the manuscript (include quotes in quotation marks "like this" to indicate direct quotes from your manuscript), or elaborate on this item by providing additional information not in the ms, or briefly explain why the item is not applicable/relevant for your study

"The intervention settings enabled recruitment until December 15, 2016, and thus the sample size would include 272 participants or truncated at the number of participants included at the end of the recruitment period—whichever was reached first"

15) A table showing baseline demographic and clinical characteristics for each group

NPT: When applicable, a description of care providers (case volume, qualification, expertise, etc.) and centers (volume) in each group

Does your paper address CONSORT subitem 15? *

Copy and paste relevant sections from the manuscript (include quotes in quotation marks "like this" to indicate direct quotes from your manuscript), or elaborate on this item by providing additional information not in the ms, or briefly explain why the item is not applicable/relevant for your study

This is presented in Table 1

15-i) Report demographics associated with digital divide issues

In ehealth trials it is particularly important to report demographics associated with digital divide issues, such as age, education, gender, social-economic status, computer/Internet/ehealth literacy of the participants, if known.

| | 1 | 2 | 3 | 4 | 5 | |
|---|---|---|---|---|---|---|
| subitem not at all important | O | O | O | O | O | essential |

Does your paper address subitem 15-i? *

Copy and paste relevant sections from the manuscript (include quotes in quotation marks "like this" to indicate direct quotes from your manuscript), or elaborate on this item by providing additional information not in the ms, or briefly explain why the item is not applicable/relevant for your study

Age, education, gender and civil status are presented in Table 1. Computer/Internet/eHealth literacy was not assessed

16) For each group, number of participants (denominator) included in each analysis and whether the analysis was by original assigned groups

16-i) Report multiple “denominators” and provide definitions

Report multiple “denominators” and provide definitions: Report N’s (and effect sizes) “across a range of study participation [and use] thresholds” [1], e.g., N exposed, N consented, N used more than x times, N used more than y weeks, N participants “used” the intervention/comparator at specific pre-defined time points of interest (in absolute and relative numbers per group). Always clearly define “use” of the intervention.

| | 1 | 2 | 3 | 4 | 5 | |
|---|---|---|---|---|---|---|
| subitem not at all important | ○ | ○ | ○ | ○ | ○ | essential |

Does your paper address subitem 16-i? *

Copy and paste relevant sections from the manuscript (include quotes in quotation marks "like this" to indicate direct quotes from your manuscript), or elaborate on this item by providing additional information not in the ms, or briefly explain why the item is not applicable/relevant for your study

Denominators for each group and each analysis is reported in Table 2

16-ii) Primary analysis should be intent-to-treat

Primary analysis should be intent-to-treat, secondary analyses could include comparing only “users”, with the appropriate caveats that this is no longer a randomized sample (see 18-i).

| | 1 | 2 | 3 | 4 | 5 | |
|---|---|---|---|---|---|---|
| subitem not at all important | ○ | ○ | ○ | ○ | ○ | essential |

Does your paper address subitem 16-ii?

Copy and paste relevant sections from the manuscript (include quotes in quotation marks "like this" to indicate direct quotes from your manuscript), or elaborate on this item by providing additional information not in the ms, or briefly explain why the item is not applicable/relevant for your study

The primary analysis is described in the Statistical Methods subsection of the Method section and reported in Table 2

17a) For each primary and secondary outcome, results for each group, and the estimated effect size and its precision (such as 95% confidence interval)

Does your paper address CONSORT subitem 17a? *

Copy and paste relevant sections from the manuscript (include quotes in quotation marks "like this" to indicate direct quotes from your manuscript), or elaborate on this item by providing additional information not in the ms, or briefly explain why the item is not applicable/relevant for your study

This is reported in Table 2

17a-i) Presentation of process outcomes such as metrics of use and intensity of use

In addition to primary/secondary (clinical) outcomes, the presentation of process outcomes such as metrics of use and intensity of use (dose, exposure) and their operational definitions is critical. This does not only refer to metrics of attrition (13-b) (often a binary variable), but also to more continuous exposure metrics such as "average session length". These must be accompanied by a technical description how a metric like a "session" is defined (e.g., timeout after idle time) [1] (report under item 6a).

| | 1 | 2 | 3 | 4 | 5 | |
|---|---|---|---|---|---|---|
| subitem not at all important | ○ | ○ | ○ | ○ | ○ | essential |

Does your paper address subitem 17a-i?

Copy and paste relevant sections from the manuscript (include quotes in quotation marks "like this" to indicate direct quotes from your manuscript), or elaborate on this item by providing additional information not in the ms, or briefly explain why the item is not applicable/relevant for your study

Adherence to the IWT intervention is presented in Multimedia Appendix 4 and adherence to the IWT protocol (repeated cycles of 3-minute slow walking and 3-minute fast walking) is presented in Multimedia Appendix 3

17b) For binary outcomes, presentation of both absolute and relative effect sizes is recommended

Does your paper address CONSORT subitem 17b? *

Copy and paste relevant sections from the manuscript (include quotes in quotation marks "like this" to indicate direct quotes from your manuscript), or elaborate on this item by providing additional information not in the ms, or briefly explain why the item is not applicable/relevant for your study

Both absolute and relative effect sizes are reported for MVPA responders, SF-12 PCS responders, and SF-12 MCS responders in Table 2

18) Results of any other analyses performed, including subgroup analyses and adjusted analyses, distinguishing pre-specified from exploratory

Does your paper address CONSORT subitem 18? *

Copy and paste relevant sections from the manuscript (include quotes in quotation marks "like this" to indicate direct quotes from your manuscript), or elaborate on this item by providing additional information not in the ms, or briefly explain why the item is not applicable/relevant for your study

Intention-to-treat analysis of changes from beseline to 12-week intermediate assessment is reported in Multimedia Appendix 6. Sensitivity analyses are reported in Multimedia Appendix 7. Subgroup analyses are reported in Figure 3 and Multimedia Appendix 9. Post hoc linear regression analyses are reported in Multimedia Appendix 8.

18-i) Subgroup analysis of comparing only users

A subgroup analysis of comparing only users is not uncommon in ehealth trials, but if done, it must be stressed that this is a self-selected sample and no longer an unbiased sample from a randomized trial (see 16-iii).

| | 1 | 2 | 3 | 4 | 5 | |
|---|---|---|---|---|---|---|
| subitem not at all important | ○ | ○ | ○ | ○ | ○ | essential |

Does your paper address subitem 18-i?

Copy and paste relevant sections from the manuscript (include quotes in quotation marks "like this" to indicate direct quotes from your manuscript), or elaborate on this item by providing additional information not in the ms, or briefly explain why the item is not applicable/relevant for your study

Such a subgroup analysis was not performed

19) All important harms or unintended effects in each group
(for specific guidance see CONSORT for harms)

Does your paper address CONSORT subitem 19? *

Copy and paste relevant sections from the manuscript (include quotes in quotation marks "like this" to indicate direct quotes from your manuscript), or elaborate on this item by providing additional information not in the ms, or briefly explain why the item is not applicable/relevant for your study

"No adverse events or harms were reported to the health professionals."

19-i) Include privacy breaches, technical problems

Include privacy breaches, technical problems. This does not only include physical "harm" to participants, but also incidents such as perceived or real privacy breaches [1], technical problems, and other unexpected/unintended incidents. "Unintended effects" also includes unintended positive effects [2].

| | 1 | 2 | 3 | 4 | 5 | |
|---|---|---|---|---|---|---|
| subitem not at all important | ○ | ○ | ○ | ○ | ○ | essential |

Does your paper address subitem 19-i?

Copy and paste relevant sections from the manuscript (include quotes in quotation marks "like this" to indicate direct quotes from your manuscript), or elaborate on this item by providing additional information not in the ms, or briefly explain why the item is not applicable/relevant for your study

The number of participants expressing technical problems with the app are reported in Multimedia Appendix 5. No privacy breaches was detected.

19-ii) Include qualitative feedback from participants or observations from staff/researchers

Include qualitative feedback from participants or observations from staff/researchers, if available, on strengths and shortcomings of the application, especially if they point to unintended/unexpected effects or uses. This includes (if available) reasons for why people did or did not use the application as intended by the developers.

| | 1 | 2 | 3 | 4 | 5 | |
|---|---|---|---|---|---|---|
| subitem not at all important | ○ | ○ | ○ | ○ | ○ | essential |

Does your paper address subitem 19-ii?

Copy and paste relevant sections from the manuscript (include quotes in quotation marks "like this" to indicate direct quotes from your manuscript), or elaborate on this item by providing additional information not in the ms, or briefly explain why the item is not applicable/relevant for your study

The only available data on feedback related to the application is reported in Multimedia Appendix 5

DISCUSSION

22) Interpretation consistent with results, balancing benefits and harms, and considering other relevant evidence

NPT: In addition, take into account the choice of the comparator, lack of or partial blinding, and unequal expertise of care providers or centers in each group

22-i) Restate study questions and summarize the answers suggested by the data, starting with primary outcomes and process outcomes (use)

Restate study questions and summarize the answers suggested by the data, starting with primary outcomes and process outcomes (use).

| | 1 | 2 | 3 | 4 | 5 | |
|---|---|---|---|---|---|---|
| subitem not at all important | ○ | ○ | ○ | ○ | ○ | essential |

Does your paper address subitem 22-i? *

Copy and paste relevant sections from the manuscript (include quotes in quotation marks "like this" to indicate direct quotes from your manuscript), or elaborate on this item by providing additional information not in the ms, or briefly explain why the item is not applicable/relevant for your study

"The main finding of this study was that InterWalk app–based IWT did not increase objectively measured MVPA time over 52 weeks compared with standard care among individuals with T2D referred to municipality-based lifestyle programs." and "In general, IWT adherence was low across 52 weeks, largely explaining the lacking effect on MVPA time."

22-ii) Highlight unanswered new questions, suggest future research

Highlight unanswered new questions, suggest future research.

| | 1 | 2 | 3 | 4 | 5 | |
|---|---|---|---|---|---|---|
| subitem not at all important | ○ | ○ | ○ | ○ | ○ | essential |

Does your paper address subitem 22-ii?

Copy and paste relevant sections from the manuscript (include quotes in quotation marks "like this" to indicate direct quotes from your manuscript), or elaborate on this item by providing additional information not in the ms, or briefly explain why the item is not applicable/relevant for your study

"Further research is needed to identify optimal implementation of digital support for PA adherence and adoption among individuals with T2D."

20) Trial limitations, addressing sources of potential bias, imprecision, and, if relevant, multiplicity of analyses

20-i) Typical limitations in ehealth trials

Typical limitations in ehealth trials: Participants in ehealth trials are rarely blinded. Ehealth trials often look at a multiplicity of outcomes, increasing risk for a Type I error. Discuss biases due to non-use of the intervention/usability issues, biases through informed consent procedures, unexpected events.

| | 1 | 2 | 3 | 4 | 5 | |
|---|---|---|---|---|---|---|
| subitem not at all important | ○ | ○ | ○ | ○ | ○ | essential |

Does your paper address subitem 20-i? *

Copy and paste relevant sections from the manuscript (include quotes in quotation marks "like this" to indicate direct quotes from your manuscript), or elaborate on this item by providing additional information not in the ms, or briefly explain why the item is not applicable/relevant for your study

Limitations to the study is discussed in third paragraph of the Discussion section

21) Generalisability (external validity, applicability) of the trial findings

NPT: External validity of the trial findings according to the intervention, comparators, patients, and care providers or centers involved in the trial

21-i) Generalizability to other populations

Generalizability to other populations: In particular, discuss generalizability to a general Internet population, outside of a RCT setting, and general patient population, including applicability of the study results for other organizations

| | 1 | 2 | 3 | 4 | 5 | |
|---|---|---|---|---|---|---|
| subitem not at all important | ○ | ○ | ○ | ○ | ○ | essential |

Does your paper address subitem 21-i?

Copy and paste relevant sections from the manuscript (include quotes in quotation marks "like this" to indicate direct quotes from your manuscript), or elaborate on this item by providing additional information not in the ms, or briefly explain why the item is not applicable/relevant for your study

"Further, the sample representativeness was limited. First, individuals of low socioeconomic status are less likely to be referred to municipality-based lifestyle programs [50]. Second, choosing to participate in an exercise program indicates a will to change PA behavior, whereas rejecting participation may not. Third, as Danish language was used during assessment (eg, questionnaires) and exercise programs, non-Danish speaking individuals were precluded from participation. Altogether, this sample may underrepresent the most vulnerable individuals with T2D. Further, our sample was more physically active at baseline than the general US population [51] and individuals with T2D in a previous study [52]. This indicates potential ceiling effect of MVPA at baseline and thus selection bias by exclusion of less physically active individuals from this sample."

21-ii) Discuss if there were elements in the RCT that would be different in a routine application setting

Discuss if there were elements in the RCT that would be different in a routine application setting (e.g., prompts/reminders, more human involvement, training sessions or other co-interventions) and what impact the omission of these elements could have on use, adoption, or outcomes if the intervention is applied outside of a RCT setting.

| | 1 | 2 | 3 | 4 | 5 | |
|---|---|---|---|---|---|---|
| subitem not at all important | ○ | ○ | ○ | ○ | ○ | essential |

Does your paper address subitem 21-ii?

Copy and paste relevant sections from the manuscript (include quotes in quotation marks "like this" to indicate direct quotes from your manuscript), or elaborate on this item by providing additional information not in the ms, or briefly explain why the item is not applicable/relevant for your study

The intervention was implemented in a municipality-based health care setting with human involvemenet and co-interventions and thus, was very close to a routine application setting.

OTHER INFORMATION

23) Registration number and name of trial registry

Does your paper address CONSORT subitem 23? *

Copy and paste relevant sections from the manuscript (include quotes in quotation marks "like this" to indicate direct quotes from your manuscript), or elaborate on this item by providing additional information not in the ms, or briefly explain why the item is not applicable/relevant for your study

"The study was approved by the scientific ethical committee of the Capital Region of Denmark (H-1-2014-074) and registered at clinicaltrials.gov (NCT02341690)."

24) Where the full trial protocol can be accessed, if available

Does your paper address CONSORT subitem 24? *

Cite a Multimedia Appendix, other reference, or copy and paste relevant sections from the manuscript (include quotes in quotation marks "like this" to indicate direct quotes from your manuscript), or elaborate on this item by providing additional information not in the ms, or briefly explain why the item is not applicable/relevant for your study

"The original protocol has been published [23],"

25) Sources of funding and other support (such as supply of drugs), role of funders

Does your paper address CONSORT subitem 25? *

Copy and paste relevant sections from the manuscript (include quotes in quotation marks "like this" to indicate direct quotes from your manuscript), or elaborate on this item by providing additional information not in the ms, or briefly explain why the item is not applicable/relevant for your study

Sources of funding is reported in the Acknowledgements section

X27) Conflicts of Interest (not a CONSORT item)

X27-i) State the relation of the study team towards the system being evaluated

In addition to the usual declaration of interests (financial or otherwise), also state the relation of the study team towards the system being evaluated, i.e., state if the authors/evaluators are distinct from or identical with the developers/sponsors of the intervention.

| | 1 | 2 | 3 | 4 | 5 | |
|---|---|---|---|---|---|---|
| subitem not at all important | ○ | ○ | ○ | ○ | ○ | essential |

Does your paper address subitem X27-i?

Copy and paste relevant sections from the manuscript (include quotes in quotation marks "like this" to indicate direct quotes from your manuscript), or elaborate on this item by providing additional information not in the ms, or briefly explain why the item is not applicable/relevant for your study

"we developed a mock-up of the app and invited persons with T2D to provide their initial feedback. Following the development of the beta-version of the app, 3 iterations of user feedback were incorporated into the design before releasing the version used in this study (versions 8 and 9) [20]."

About the CONSORT EHEALTH checklist

As a result of using this checklist, did you make changes in your manuscript? *

- ○ yes, major changes
- ○ yes, minor changes
- ◉ no

What were the most important changes you made as a result of using this checklist?

Dit svar

How much time did you spend on going through the checklist INCLUDING making changes in your manuscript *

Several days............

As a result of using this checklist, do you think your manuscript has improved? *

- ○ yes
- ◉ no
- ○ Andet:

Would you like to become involved in the CONSORT EHEALTH group?

This would involve for example becoming involved in participating in a workshop and writing an "Explanation and Elaboration" document

- ○ yes
- ◉ no
- ○ Andet:

Ryd markering

Any other comments or questions on CONSORT EHEALTH

Dit svar

STOP - Save this form as PDF before you click submit

To generate a record that you filled in this form, we recommend to generate a PDF of this page (on a Mac, simply select "print" and then select "print as PDF") before you submit it.

When you submit your (revised) paper to JMIR, please upload the PDF as supplementary file.

Don't worry if some text in the textboxes is cut off, as we still have the complete information in our database. Thank you!

Final step: Click submit !

Click submit so we have your answers in our database!

Send

Ryd formular

Indsend aldrig adgangskoder via Google Analyse.

Dette indhold er hverken oprettet eller godkendt af Google. Rapportér misbrug - Servicevilkår - Privatlivspolitik

Google Analyse
