# Supplementary material for: The Effects of a Lifestyle Intervention Supported by the InterWalk Smartphone App on Increasing Physical Activity Among Persons With Type 2 Diabetes: Parallel-Group, Randomized Trial
Source: JMIR Mhealth Uhealth. 2022 Sep 28;10(9):e30602. doi: 10.2196/30602 (PMC9557767; doi:10.2196/30602)
Supplement: Supplementary file 11 [file mhealth_v10i9e30602_app10.docx]

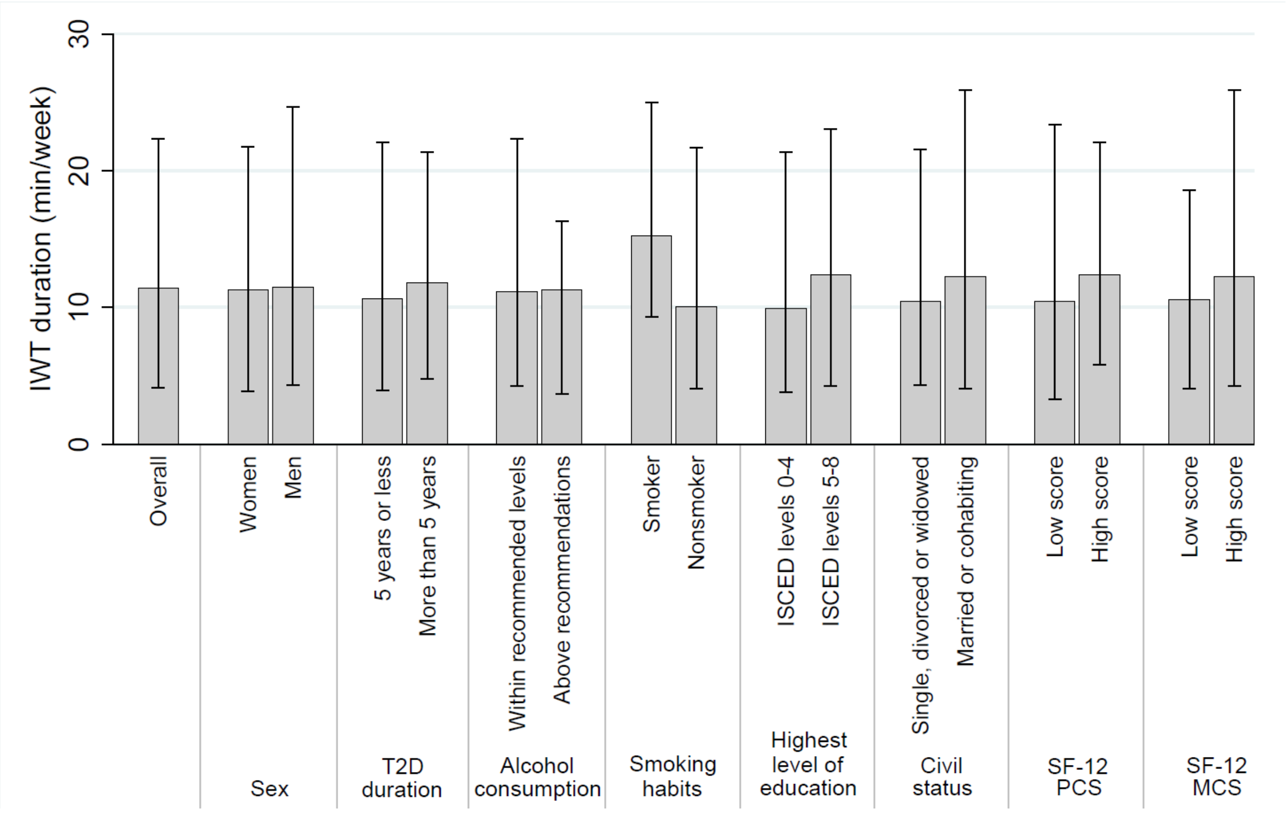

IWT duration (min/week)
0
10
20
30
Overall
Women
Men
5 years or less
More than 5 years
Within recommended levels
Above recommendations
Smoker
Nonsmoker
ISCED levels 0-4
ISCED levels 5-8
Single, divorced or widowed
Married or cohabiting
Low score
High score
Low score
High score
Sex
T2D duration
Alcohol consumption
Smoking habits
Highest level of education
Civil status
SF-12 PCS
SF-12 MCS
