# Supplementary material for: The Effects of a Lifestyle Intervention Supported by the InterWalk Smartphone App on Increasing Physical Activity Among Persons With Type 2 Diabetes: Parallel-Group, Randomized Trial
Source: JMIR Mhealth Uhealth. 2022 Sep 28;10(9):e30602. doi: 10.2196/30602 (PMC9557767; doi:10.2196/30602)
Supplement: Supplementary file 12 [file mhealth_v10i9e30602_app3.docx]

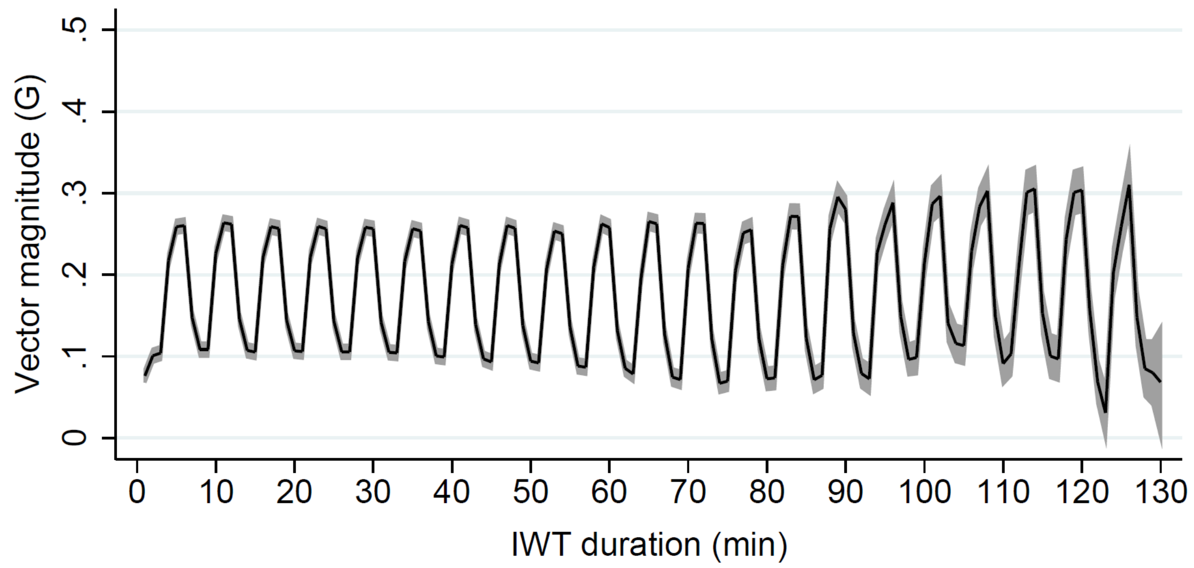

Vector magnitude (G)
0
.1
.2
.3
.4
.5
0
10
20
30
40
50
60
70
80
90
100
110
120
130
IWT duration (min)
